# Integrating machine learning and symbolic regression for predicting damage initiation in hybrid FRP bolted connections

**DOI:** 10.1038/s41598-025-02390-4

**Published:** 2025-05-27

**Authors:** Sherif Samy Sorour, Chahinaz Abdelrahman Saleh, Mostafa Shazly

**Affiliations:** 1 Department of Mechanical Engineering, The British University in Egypt, El Sherouk City, Cairo, 11837 Egypt; 2 Department of Mechanical Design and Production Engineering, Faculty of Engineering, Cairo University, Giza, 12613 Egypt

**Keywords:** Machine learning, Symbolic regression, PySR, Bolted connections, Laminated FRP composites, Mechanical engineering, Computational science, Composites

## Abstract

The increasing adoption of machine learning (ML) in fiber-reinforced polymer (FRP) composite design has led to a reliance on black-box models, which achieve high predictive accuracy but lack interpretability. Python symbolic regression (PySR) offers a solution by deriving explicit equations that reveal the governing mechanics of composite structures. This study focuses on hybrid FRP bolted connections, which are rapidly adopted in the industry but remain insufficiently addressed in academic research. To address this gap, a framework was developed to identify key design parameters and predict damage initiation loads by integrating experimental testing, finite element modeling (FEM), and ML. Feature selection and ML models analyzed the dataset, providing insights that guided PySR in deriving interpretable equations. Hybrid L-joint specimens were fabricated and tested to determine damage initiation loads, with results validating FEM models in ABAQUS. A design of experiments approach structured the dataset, and feature selection identified key factors influencing joint performance. ML models assessed dataset quality, with Huber regression emerging as the best-performing model. Based on insights from feature analysis and ML models, PySR derived a compact, interpretable equation that provided greater accuracy and deeper physical insights than the Huber model. This equation aids hybrid L-joint design by improving the understanding of damage initiation mechanics. Beyond predictive accuracy, the findings highlight the model’s scalability to different bolt sizes, equally spaced row of bolts, and stacking sequences. This study demonstrates the potential of interpretable ML in structural engineering applications, particularly for hybrid composite-metal joints, where transparent models are essential for design optimization and predictive accuracy.

## Introduction

The increasing adoption of machine learning (ML) in laminated fiber-reinforced polymer (FRP) composite design is supported by recent bibliometric analyses conducted in our prior review^1^, highlighting a sharp rise in ML-related publications since 2018. ML has been widely applied in various aspects of FRP composite research, including microstructure-property linkage^2–4^, mechanical properties prediction^5,6^, damage parameter calibration^7,8^, damage parameter prediction^9–11^, damage initiation prediction^12,13^, stiffness degradation analysis^14,15^, progressive damage analysis^16–19^, crack path detection^5^, design and optimization of FRP composites^20–22^, drilling of laminated composites^23,24^, and bolted/bonded connections^25,26^. A strong reliance on artificial neural networks (ANNs), including deep learning, and ensemble models such as Random Forest (RF) and XGBoost has been widely reported in FRP composite research, including but not limited to these studies. Despite achieving high predictive accuracy, these black-box models pose significant interpretability challenges in engineering design, where understanding variable relationships is crucial for informed decision-making.

Symbolic regression, particularly through Python Symbolic Regression (PySR), provides an alternative by deriving explicit equations directly from data. Unlike traditional black-box models, PySR generates interpretable mathematical relationships, revealing the governing mechanics of composite behavior. PySR has demonstrated superior performance over conventional symbolic regression techniques^27^. PySR can also interpret black-box models by re-expressing them into simplified symbolic expressions, this capability was demonstrated in the 2024 study involving MIT, CERN, and Princeton University^28^. PySR has also been successfully applied in materials science for fatigue crack growth rate modeling^29^ and biomechanics to develop interpretable strain energy functions for human brain tissue^30^. Similar efforts to harness symbolic and nonlinear regression models for transparent mechanical behavior prediction have emerged in other civil engineering domains, such as compressive strength prediction of engineered cementitious composites using Gene Expression Programming (GEP)^31^ and nonlinear regression modeling of shape memory alloy (SMA)-confined concrete columns^32^. These studies highlight the broader demand for interpretable modeling in structural material systems. Given the critical need for transparent ML models in engineering design applications, PySR serves as both an alternative to black-box models and a tool to interpret them.

This research paper employs symbolic regression via PySR to derive an interpretable equation for damage initiation load in hybrid FRP bolted connections. These connections were chosen due to the challenges posed by their rapid industrial adoption, as demonstrated in applications such as the Airbus A350 XWB^33^, Boeing 787 Dreamliner^34,35^, the Leading Edge Aviation Propulsion (LEAP) engine^36^, BMW i-series^37^, and Audi R8 e-tron^38^, along with specific real-world projects, such as the FabHeli Project^39^ and Com-Bridge in Poland^40,41^.

Despite this widespread adoption of these joints, the EUROCOMP guidelines rely on simplified assumptions, such as zero-hole clearance and frictionless interfaces^42^. These assumptions have faced criticism for their lack of practical relevance in real-world applications. Previous studies, including the work of Turvey and Wang^42^ demonstrated that even minimal clearance and friction significantly alter stress distributions around bolted joints, challenging the applicability of EUROCOMP’s simplified method to real-world scenarios.

To address this gap, the present study focuses on hybrid GFRP-steel bolted L-joint connections, a structural configuration that remains underexplored in both academic research and current design codes. By integrating experimental testing, finite element modeling (FEM), and machine learning techniques, this work aims to provide a more realistic and interpretable framework for predicting damage initiation loads, ultimately bridging the gap between theoretical models and practical industrial applications. Furthermore, this study attempts to propose the first explicit equation that directly relates damage initiation load to key design parameters in hybrid GFRP-steel L-joint bolted connections, offering a concise and interpretable formulation that can aid in structural optimization and engineering decision-making.

## Methodology overview

This study integrates experimental testing, finite element modeling (FEM), and symbolic regression to derive interpretable equations for predicting damage initiation loads in hybrid L-joint connections. Experimental tests were conducted on fabricated GFRP-steel bolted L-joint specimens, with load–displacement data collected to characterize mechanical behavior and validate FEM simulations developed using ABAQUS.

A hybrid Design of Experiments (DoE) approach, combining Central Composite Design (CCD) and Box-Behnken Design (BBD), structured the dataset, ensuring comprehensive exploration of non-linear interactions between geometric parameters. Feature selection techniques were applied to identify critical variables influencing joint performance. Multiple ML models were then trained to validate the dataset’s predictive capability, with the best-performing model serving as a benchmark for comparison with PySR and guiding the selection of its loss function.

The symbolic regression model incorporates the clearance, tightening torque, and dimensionless geometric ratios (e.g., E/L, W/D, E/D), offering explicit and interpretable relationships between geometry and damage initiation load. By incorporating hole clearance and friction into the FEM model, this framework offers a realistic model that bridges the gap between theoretical analysis and practical engineering applications, especially for configurations not adequately addressed by existing standards.

## Experimental procedure

### Hybrid L-joint fabrication

Hybrid L-joint specimens were fabricated from unidirectional E-glass/epoxy composite laminates using a [0°]₄ layup configuration. A commercial-grade, non-woven E-glass mat (UNI E1050 M50, supplied by Selcom) was combined with a two-component epoxy resin system (Araldite® LY1564, supplied by Huntsman). The fabrication was carried out using the vacuum-assisted resin infusion (VARI) process (Appendix A, Fig. A1). The process commenced with thorough mold cleaning and the precise application of a wax release agent. Four layers of unidirectional glass fiber were carefully stacked onto the mold, and a vacuum bag was secured around the assembly. With the vacuum pump in operation, air was extracted, drawing resin uniformly through designated feeding lines while the excess resin was simultaneously removed via a vacuum line. This procedure resulted in the formation of a composite L-shaped part.

**Fig. 1:**
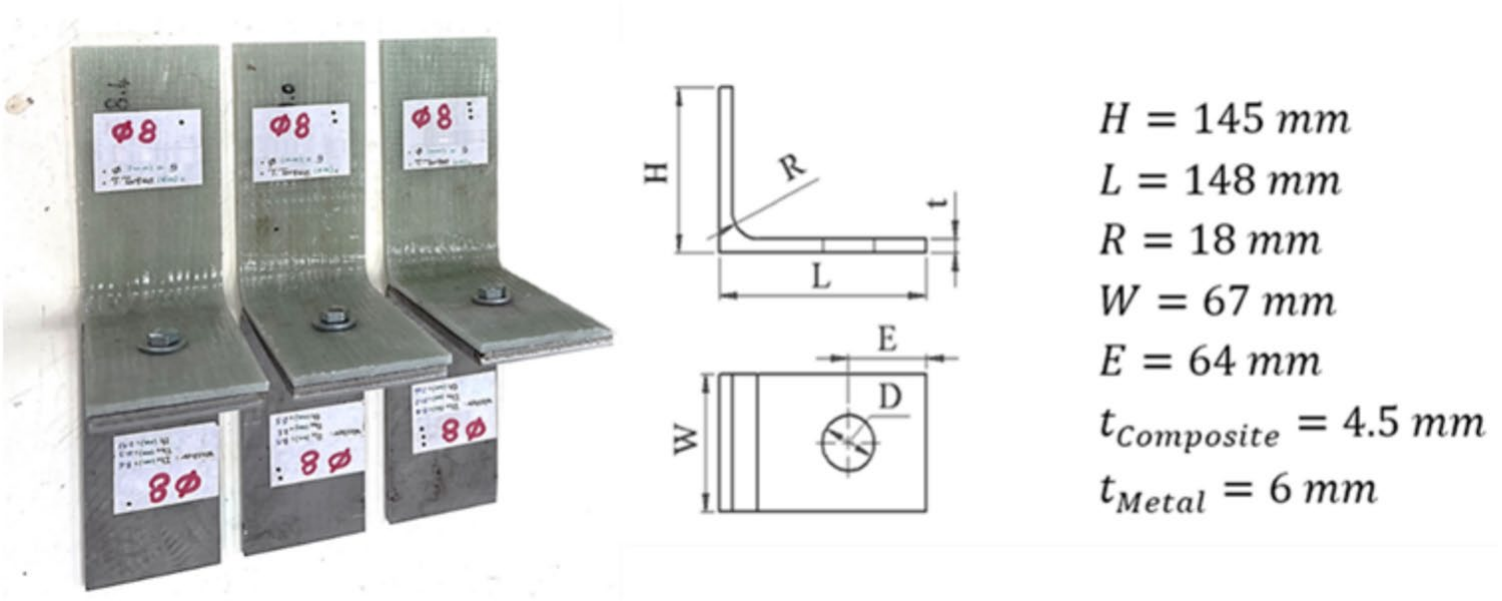
Assembled L-joints hybrid connections with dimensional specifications.

The cured composite part was trimmed and cut to the required dimensions using Pedrini M940 CNC machine. The composite periphery was trimmed to remove irregular outer edges and then divided into individual samples. These operations were executed with a feed rate of 150 mm/min and a cutting speed of 2394 m/min, with a lubricant applied during machining to ensure precision and minimize thermal effects (Appendix A, Fig. A2). Visual inspections confirmed that any damage to the specimens was negligible (Appendix A, Fig. A3).

**Fig. 2:**
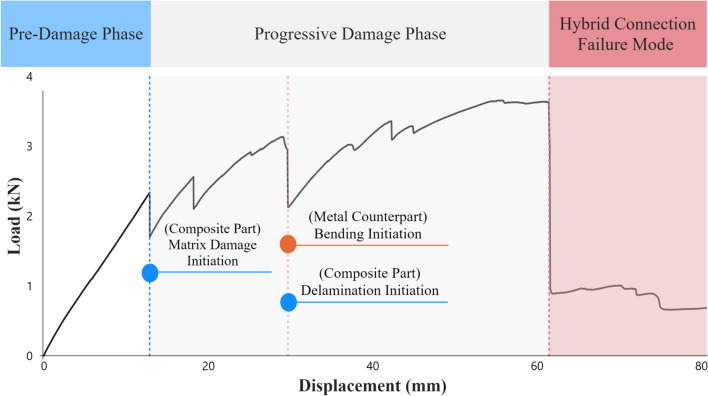
Illustration of load–displacement curve phases for the tested hybrid connections.

**Fig. 3:**
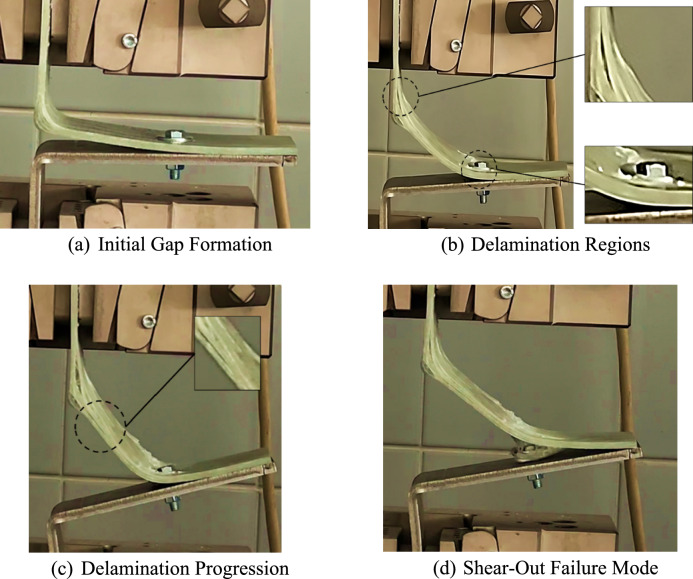
Reported observations during the load–displacement test of hybrid connections.

Subsequently, bolt holes were machined using an Waterjet Sweden NC 3020 (Appendix A, Fig. A4). The waterjet process employed GMA garnet 80 abrasive at a pressure of 3800 bar, a flow rate of 300 gm/min, a nozzle-to-workpiece distance of 2 mm, and an average cutting feed rate of 100 mm/min, ensuring that the holes met the prescribed dimensions for medium-fit M8 bolts maintaining alignment with ISO 273-1979 (2019) guidelines for M8 medium-fit bolts^43^.

**Fig. 4:**
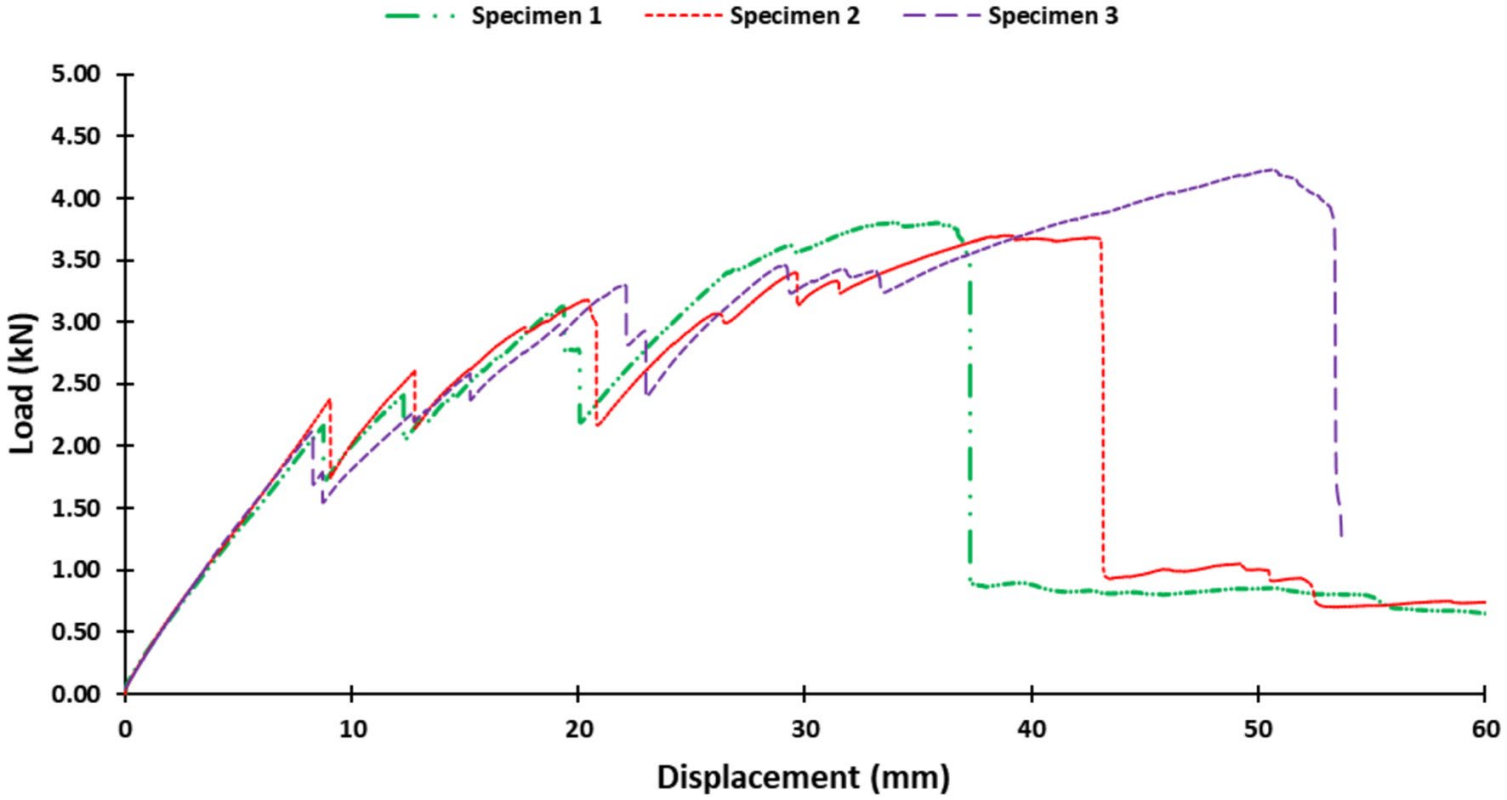
Load–displacement curves for the tested specimens.

Visual inspections revealed no significant damage, with minimal delamination observed at the hole entry across the inlet, outlet, and through-thickness regions. Backlight imaging was employed to quantify delamination, confirming that any damage was confined within 0.41 mm from the hole perimeter and limited to specific angular intervals. Detailed inspection results are provided in Appendix A.

The steel L-joint components were fabricated from Steel 37 sheets, maintaining a thickness of 6 mm to delay bending and focus on composite deformation. The manufacturing process included laser cutting followed by 90° bending. Bolted connections were assembled using grade 8.8 steel bolts and washers, with a torque of 28.8 N·m applied according to ISO 898-1 specifications^44^, the final assembly is provided in Fig. 1.

### Experimental testing

#### Load–displacement testing

The mechanical response of hybrid L-joints was evaluated through load–displacement testing, performed under quasi-static conditions adhering to ASTM D3039/D3039M-08 standards^45^. The load–displacement curve, illustratively presented in Fig. 2, highlights three distinct behaviors in hybrid connections:

- Pre-Damage Phase: During the initial stage, the transition from linearly elastic behavior to the onset of damage in the composite component is observed. The curve starts with a steep linear segment, characterizing the initial elastic response. Simultaneously, a gap forms between the composite and metal components, as depicted in Fig. 3a, leading eventually to damage initiation in the composite matrix due to tension near the bolted region at the interface between the composite and the metal part.
- Progressive Damage Propagation Phase: Following the Pre-Damage Phase, this subsequent stage reveals the progressive propagation of damage within the composite material, appearing as distinct fluctuations in the load–displacement curve. Notably, each fluctuation signifies the occurrence of a new damage initiation or the propagation of existing damage. This phase highlights the initiation of delamination damage within the bolt region, accompanied by the start of metal bending of the metal counterpart. Delamination then initiates in the fillet region (Fig. 3b) and, with increasing displacement, extends from the fillet towards the bolt area (Fig. 3c).
- Failure Mode of Hybrid Connection: The final phase ends in a bolted connection failure mode, identified as out-of-plane shear-out, as illustrated in Fig. 3d.

The load–displacement curves for the samples, presented in Fig. 4, were analyzed to determine the damage initiation loads, summarized in Table 1. From this analysis, the mean damage initiation load was calculated to be 2.22 kN. The calculated standard deviation is approximately 0.132 kN, corresponding to a coefficient of variation of about 5.96%, which indicates a relatively low variability in the damage initiation loads among the specimens.

**Table 1:** Summary of damage initiation loads for L-joint specimens.

Specimen	Damage initiation load (kN)
1	2.17
2	2.37
3	2.12
Mean	2.22

#### Burn-out test for fiber volume fraction

The fiber volume fraction and void content of the GFRP laminates were assessed using the burn-out method, following ASTM D3171-22^46^. Specimens were cut to a uniform size of 2.5 × 2.5 cm and subjected to controlled heating at 590 °C for two hours. Post-heating, mass loss was used to calculate fiber and matrix fractions. The results were analyzed statistically as provided in Table 2 which indicates a uniform fiber distribution, moderate epoxy content variation, and limited void presence among samples but with lower variability than the epoxy content.

**Table 2:** Statistical analysis of GFRP laminate volume fraction and void content.

Parameter	Mean	SD
Fiber volume fraction	50.24	0.80
Matrix volume fraction	45.62	1.86
Void content	4.14	1.06

## Finite element modelling

### FEA software and solver

The finite element analysis was conducted using ABAQUS 2020/Explicit Solver. This section details the modeling techniques, material properties, and boundary conditions employed to simulate the load–displacement response of composite and metallic L-joint hybrid connections.

### Parts modeling and material properties

The composite L-joint was modeled using a layer-by-layer approach with four layers, each 1.125 mm thick, employing 8-noded continuum shell elements (SC8R) with second-order accuracy, enhanced hourglass control, and three integration points. A 1.5 mm global mesh size was applied and refined to 4 mm in clamped regions, resulting in approximately 15,000 elements per layer. Mesh sensitivity trials were conducted to ensure model accuracy. Material properties for the unidirectional GFRP and Hashin’s damage initiation parameters are detailed in Tables 3 and 4.

**Table 3:** Physical and mechanical properties of GFRP/epoxy unidirectional lamina^47^.

Property	Value
Density ρ (kg/m^3^)	1773
Longitudinal modulus E_11_ (GPa)	42
Transverse moduli E_22_ = E_33_(GPa)	10.33
Poisson’s ratio ν_12_ = ν_13_	0.238
Poisson’s ratio ν_23_	0.37
Longitudinal shear moduli G_12_ = G_13_ (GPa)	4.1
Transverse shear modulus G_23_ (GPa)	3.77

**Table 4:** Hashin’s damage initiation parameters for GFRP/epoxy^48^.

Property	Value
Longitudinal tensile strength X^T^ (MPa)	867
Longitudinal compressive strength X^C^ (MPa)	800
Transverse tensile strength Y^T^ (MPa)	97
Transverse compressive strength Y^C^ (MPa)	97
In-plane shear strength S_12_ (MPa)	60
Interlaminar shear strength S_13_ (MPa)	60
Interlaminar shear strength S_23_ (MPa)	60

The metallic counterpart, modeled as shown in Fig. 1, used 32,000 SC8R elements with a 2 mm global mesh size and 4 mm mesh refinement in clamped regions. A bilinear material model was applied, with properties listed in Table 5.

**Table 5:** Physical and mechanical properties of ST37 steel^49,50^.

Property	Value
Density ρ (kg/m^3^)	7850
Elastic modulus E (GPa)	210
Poisson’s ratio ν	0.3
Yield strength σ_y_ (MPa)	235
Ultimate tensile strength σ_UTS_ (MPa)	340
Elongation	0.26

The hexagonal bolt, nut, and washer were modeled according to ISO 4014 standards, using C3D8R solid elements with a 0.6 mm mesh size. A bilinear material model was applied for all components, resulting in approximately 20,000 elements for the bolt, 2900 for the nut, and 4200 for the washer, with material properties provided in Tables 6 and 7.

**Table 6:** Physical and mechanical properties of grade 8.8 bolts^51^.

Property	Value
Density ρ (kg/m^3^)	7800
Elastic modulus E (GPa)	210
Poisson’s ratio ν	0.3
Yield strength σ_y_ (MPa)	640
Ultimate tensile strength σ_UTS_ (MPa)	800
Elongation	0.12

**Table 7:** Physical and mechanical properties of low-carbon steel washers^52^.

Property	Value
Density ρ (kg/m^3^)	7872
Elastic modulus E (GPa)	206
Poisson’s ratio ν	0.29
Yield strength σ_y_ (MPa)	235
Ultimate tensile strength σ_UTS_ (MPa)	360
Elongation	0.2

### Cohesive surfaces definition

Delamination damage initiation is evaluated through the quadratic traction criterion, which requires defining Normal Interfacial Strength, 1st and 2nd Shear Interfacial Strengths, along with Normal Fracture Energy (G_IC_), 1st and 2nd Shear Fracture Energy (G_IIC_, G_IIIC_). The progression of delamination is modeled using a linear softening cohesive zone model, with the mixed-mode behavior governed by the power law criterion. Table 8 lists the traction–separation parameters for cohesive damage definition in ABAQUS.

**Table 8:** Cohesive surface damage initiation and evolution parameters^48^.

Property	Value
Normal interfacial strength Ti (MPa)	37
1st shear interfacial strength S1 (MPa)	45
2nd shear interfacial strength S2 (MPa)	45
Normal fracture energy G_IC_ (J/m^2^)	1300
1st shear fracture energy G_IIC_ (J/m^2^)	2150
2nd shear fracture energy G_IIIC_ (J/m^2^)	2150
Power law exponent	1.45

Cohesive surface interactions in ABAQUS are modeled using a spring-based approach, where adjacent faces remain initially connected. This required defining penalty stiffness coefficients in the normal (K_nn_), first shear (K_ss_), and third shear (K_tt_) directions. Since these coefficients are not physically measurable, they must be calibrated to ensure numerical stability in ABAQUS^53,54^. Based on literature recommendations^55–58^, numerous trials were conducted, which resulted in identifying the penalty stiffness coefficients in our study as K_nn_ = 8 × 10^14^ N/m^3^, with K_ss_ = K_tt_ = 10^14^ N/m^3^.

### Bolt tightening torque and load–displacement simulation

**Fig. 5:**
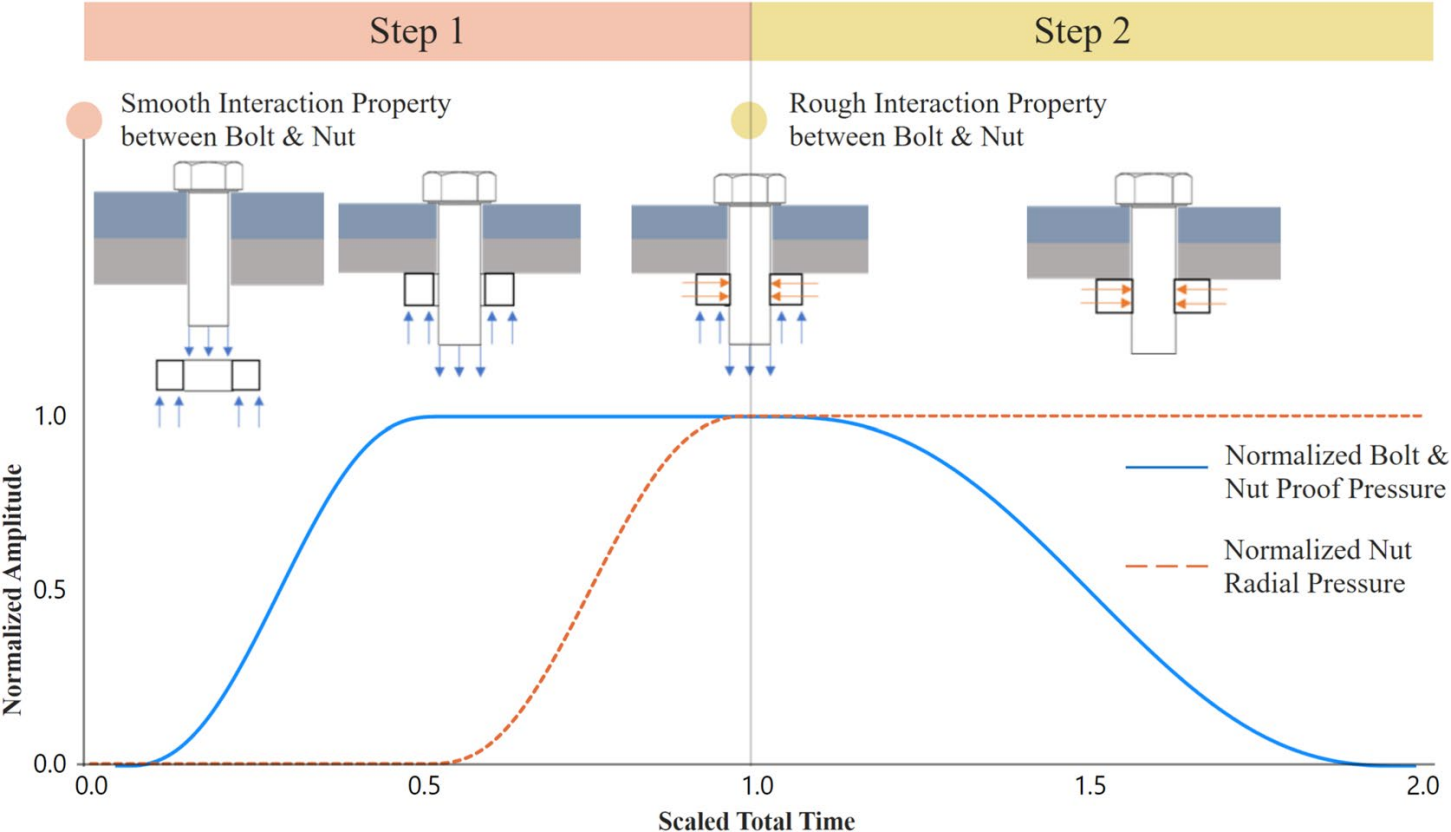
Tightening torque modelling approach.

Simulating bolt preload in ABAQUS/Standard using the Bolt Load module presented convergence challenges when combined with cohesive surface interactions, limiting its reliability for hybrid bolted joint simulations. To address this, a two-step approach was developed (Fig. 5). Initially, a proof pressure equivalent to the ISO 898-1 proof load^44^ was applied to simulate bolt tightening, with the bolt-nut interaction initially set as smooth to ensure proper load transfer. Once the proof pressure was achieved, radial pressure was applied to the nut’s inner cylindrical surface, simulating the locking effect before transitioning to the next simulation step.

In the subsequent step, the bolt-nut interaction was modified from smooth to rough contact, ensuring both components acted as a single unit. To mitigate potential element distortion during this transition, the Penalty Contact Method with finite sliding was employed, allowing slight nodal displacement due to elastic recovery. A calibrated radial pressure corresponding to a 0.045 mm interference fit ensured numerical stability while maintaining realistic stress distributions in the bolt shank.

**Fig. 6:**
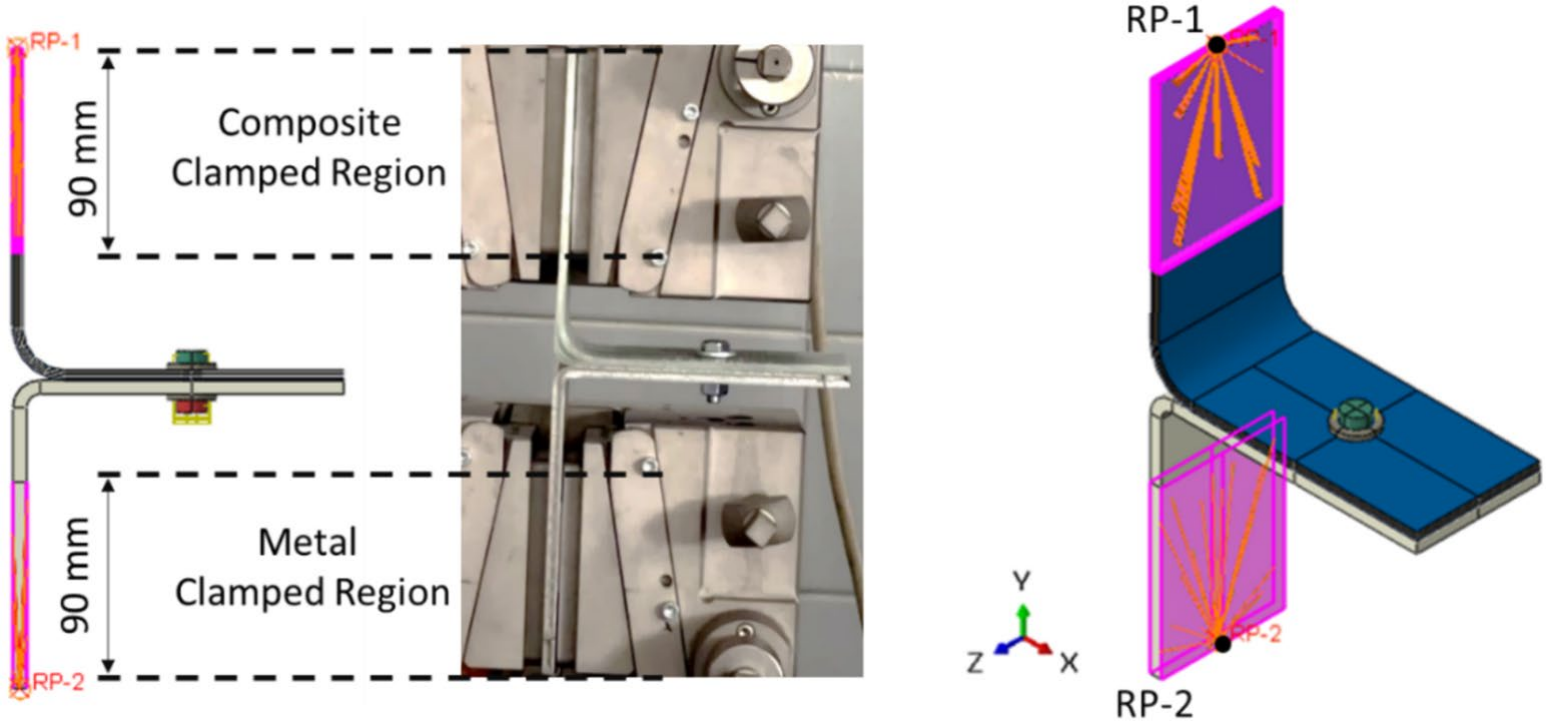
Illustration of clamped regions and reference point configurations for composite L-joint and metal counterpart.

The load–displacement curve was generated by defining two reference points (RP-1 and RP-2) for tracking displacement and load over time (Fig. 6). The composite L-joint was coupled to RP-1, which was subjected to displacement along the Y-axis with a smooth step profile, while RP-2, attached to the metal component, remained fully constrained. This setup enabled the construction of a high-resolution load–displacement curve with 400 data points, capturing detailed mechanical behavior.

### Boundary conditions and contact definition

The Boundary conditions and contact definitions were applied sequentially in three steps to accurately replicate the mechanical behavior of the hybrid L-joint system. Throughout these steps, a uniform friction coefficient of 0.25 was assigned to all contact surfaces, ensuring realistic interaction between the composite and metallic parts. Cohesive surfaces were defined using ABAQUS’s general contact algorithm, enabling continuous monitoring of damage initiation and delamination progression throughout the simulation steps.

In the first stage, the tightening torque simulation, the metallic L-joint was fully constrained at reference point RP-2, while the composite L-joint was kinematically coupled to RP-1, limiting displacement along the Y-axis. A frictionless surface-to-surface contact definition facilitated smooth load transfer during the application of proof pressure.

In the second stage, the bolt locking simulation, once the proof pressure was achieved, the bolt-nut interaction was modified from smooth to rough contact. A radial pressure was applied to the inner cylindrical surface of the nut to maintain preload. The penalty contact method with finite sliding ensured stable stress distribution, preventing element distortion during the tightening process.

In the final stage, the load–displacement simulation, a displacement-controlled load was applied to RP-1 to simulate experimental conditions, while RP-2 remained fully constrained throughout the simulation.

### FEA verification and validation

#### Energy balance analysis

**Fig. 7:**
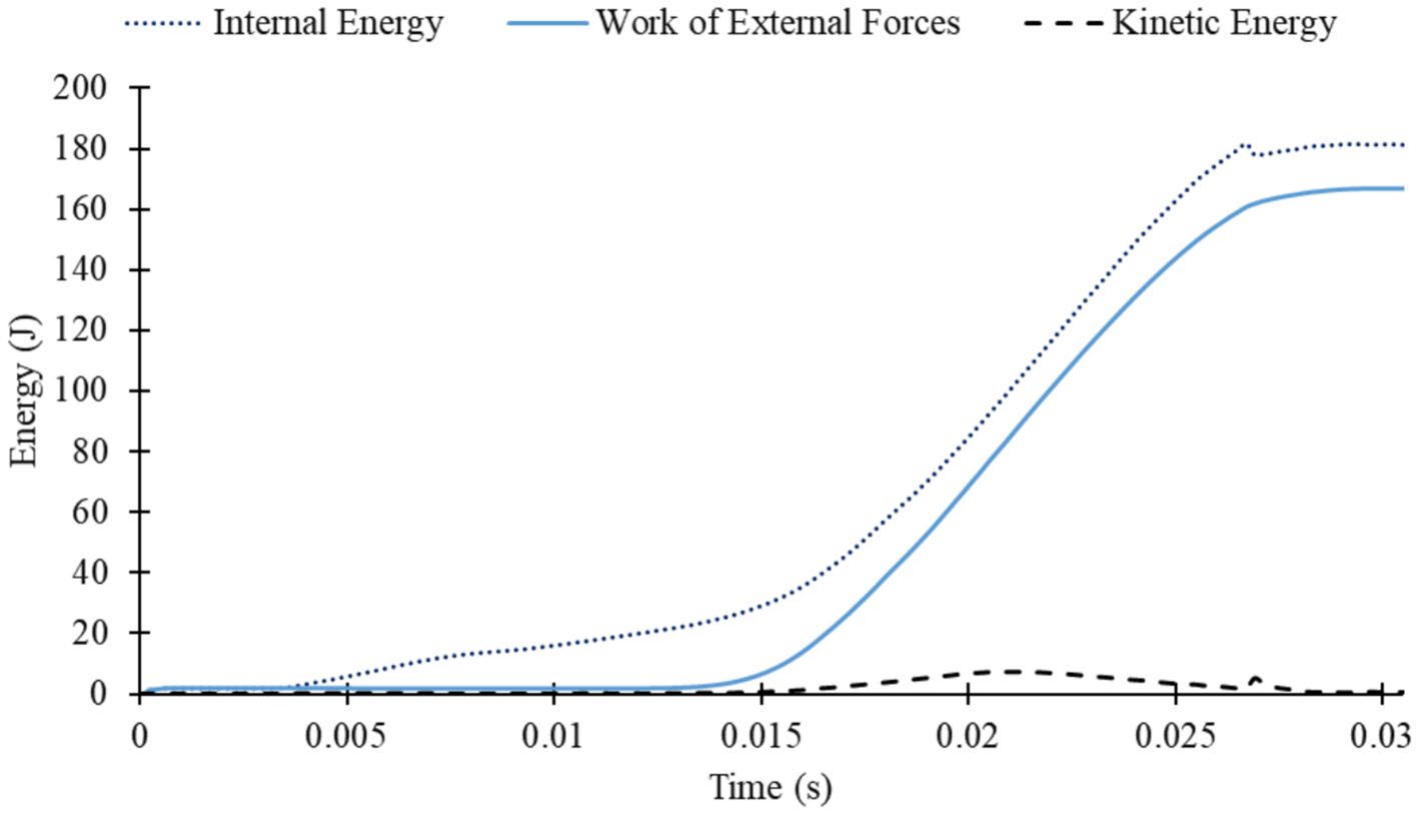
Energy output in M8 bolted L-joint simulation.

As shown in Table 9, step times for the finite element analysis were selected following ABAQUS/Explicit documentation^59^ for quasi-static simulations, which require an Energy Balance Analysis. This involves monitoring Internal Energy (ALLIE), Kinetic Energy (ALLKE), and External Work (ALLWK), with kinetic energy remaining below 5–10% of both internal energy and work done to confirm quasi-static behavior. For the M8 bolted specimen, the energy history outputs (Fig. 7) showed an average Kinetic-to-Internal Energy ratio of 2.92% and a Kinetic-to-Work Done ratio of 4.64%, both well within acceptable limits. These results confirm that dynamic effects were minimal, ensuring accurate quasi-static simulation behavior.

**Table 9:** Selected step time.

Tightening torque simulation	Load–displacement test simulation	
Step 1	Step 2	Step 3
0.0005 s	0.01 s	0.02 s

#### Axial stress validation in bolts

**Fig. 8:**
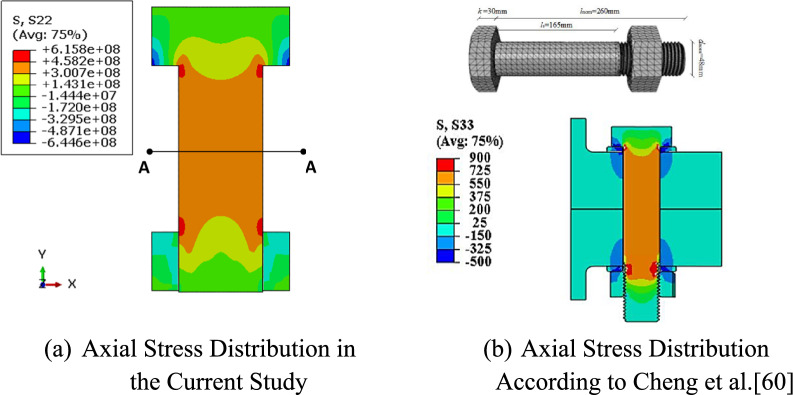
Comparative stress distribution contours in the preloaded bolt.

The axial stresses in the bolt due to tightening torque exhibit a symmetrical stress pattern along its axis. The highest stress concentrations occur under the bolt head and nut, while the shank maintains a uniform stress profile, indicating an even preload distribution (Fig. 12a). The average midsection stress (A-A) was 385 MPa, approximately 8.3% lower than the theoretical value of 420 MPa. A study by Cheng et al.^60^ modeled bolt preload using the "turn-of-nut" method, a widely adopted approach in both practical applications and numerical modeling. In their finite element model, preload was applied by rotating the nut, allowing the bolt to elongate and generate a preload force. Their results showed a similar axial stress distribution (Fig. 8a,b), with pronounced stress concentrations at the bolt interfaces, aligning with the findings of this study. While direct stress magnitude comparisons are limited due to differences in material properties and preload values, the consistent stress distribution trends validate the reliability of the approach adopted in this study.

#### Load–displacement curve validation

**Fig. 9:**
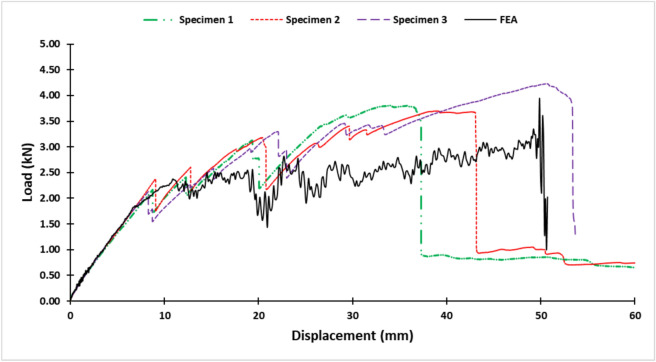
Load–displacement curves from FEA simulation and experimental results.

Figure 9 presents a comparison of the Load–Displacement curves derived from both FEA simulations and experimental data. The FEA model predicts a damage initiation load of 2.38 kN which is approximately 7.20% from the experimental mean. The FEA results captured the overall trend observed in the Load–Displacement curve. Notably, the onset of damage in the FEA model aligns within the range of the initial two fluctuations observed in the experimental Load–Displacement data, suggesting that the FEA’s estimated damage initiation load may represent an averaged response relative to these early experimental fluctuations.

**Fig. 10:**
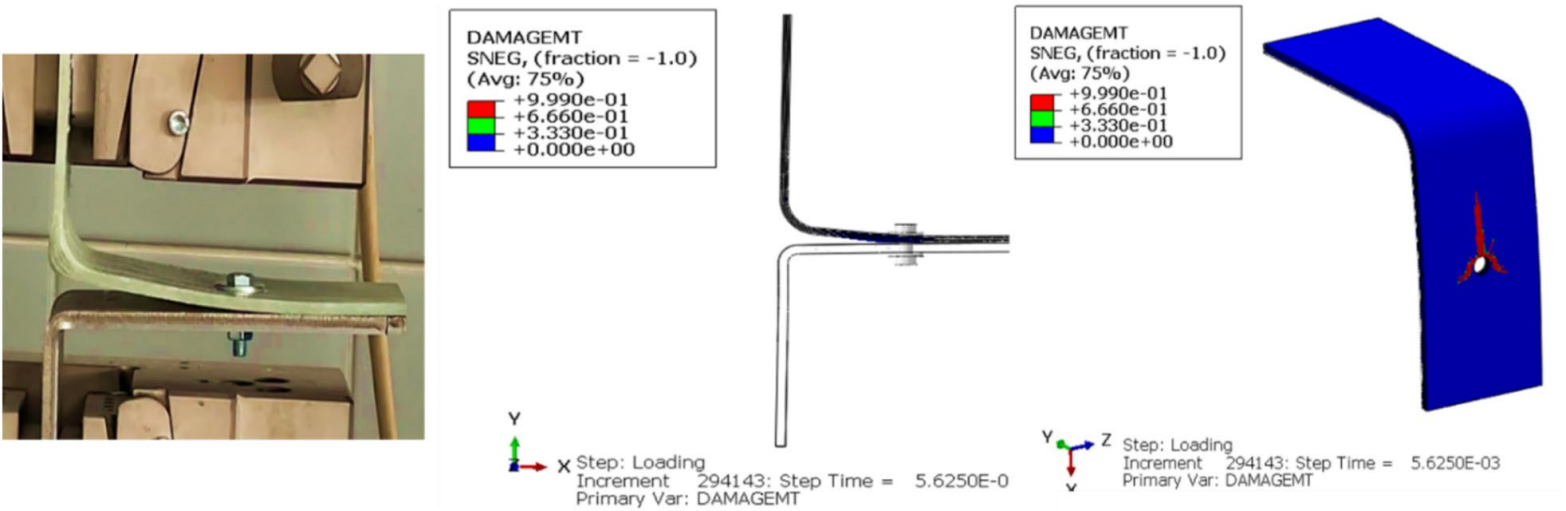
Predicted damage initiation from FEA.

**Fig. 11:**
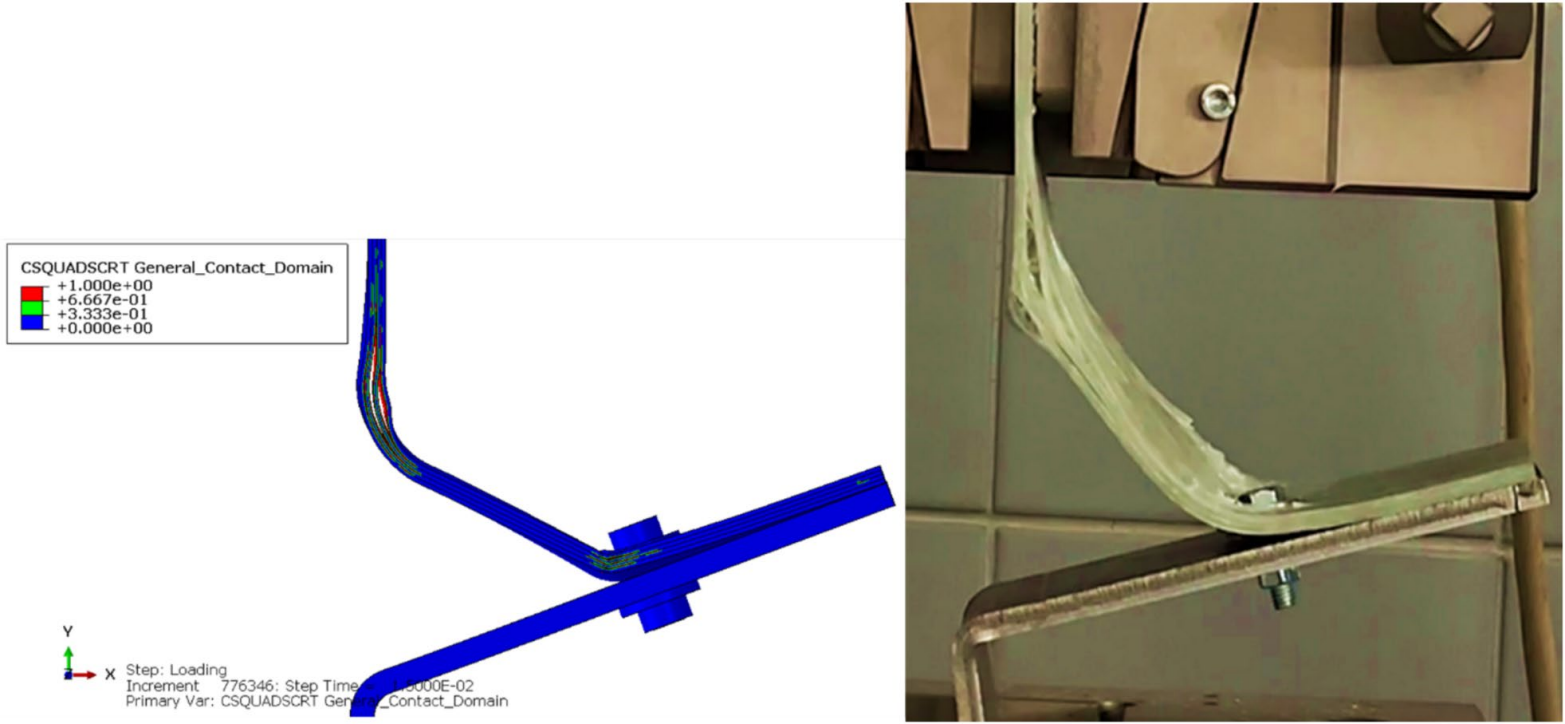
Delamination progression as predicted from FEA simulation.

The simulation predicted the composite matrix damage initiation due to tension, as shown in Fig. 10, at the step time of 5.625e-3 s. Other intralaminar damages at that step time were deemed negligible (Appendix B). The simulation predicted the delamination occurrence at the bolt region during the metal bending and predicted the fillet delamination and its subsequent progression toward the bolt area, as depicted in Fig. 11.

## Machine learning framework

### Dataset generation using design of experiments

A hybrid dataset generation approach combining Central Composite Design (CCD) and Box-Behnken Design (BBD) was used to efficiently explore the design space. CCD effectively captures quadratic effects and responses at the design space boundaries using star and center points, while BBD focuses on midpoint interactions with fewer experimental runs compared to a full factorial design. This hybrid methodology ensures the dataset captures both quadratic effects and potential higher-order interactions at an early stage of the analysis.

#### Selection of design parameters

This study focuses on six parameters related to an M8 bolt size nominal diameter (D): fillet radius (R), base length (L), edge distance (E), base width (W), tightening torque (T), and a hole size multiplier factor (φ) representing clearance (C). The clearance factor is defined by the ratio of the actual hole diameter to the nominal size of the bolt, as expressed in Eq. (1):

1 $${\varphi } = \frac{{D_{Actual} }}{D}$$

#### Constraints and criteria for dataset generation

The dataset generation process using CCD and BBD required applying constraints to ensure geometrical validity and practical relevance. All parameters, except tightening torque (T), are restricted to positive and non-zero values. Tightening torque is selected within a range defined by ISO 898-1 standards^44^ to avoid unrealistically high values. The clearance factor (φ), based on ISO 273-1979 standards^43^, the interval of this multiplier was determined to be 1 < φ ≤ 1.3 for all hole sizes. To ensure the geometric validity of the model, the base width (W), edge distance (E), and base length (L) must satisfy specific inequalities, defined by Eqs. (2)-(4):

2 $$W \ge D_{Washer}$$

3 $$E \ge (D_{Washer} )/2$$

4 $$L \ge E + R + t + (D_{Washer} )/2$$

#### Transforming design parameters into dimensional ratios

Dimensionless ratios are essential for generalizing bolted lap joint performance and enabling comparative assessments across different configurations. Key ratios include W/D (plate width-to-hole diameter), E/D (edge distance-to-hole diameter), and R/L (fillet radius-to-base length), which characterize joint geometry. Torque values are normalized for consistency, and the reciprocal of the hole size multiplier φ is used to better interpret interactions with W/D and E/D. Additionally, the E/L ratio is introduced to capture geometric variations unique to L joints, as E/D alone does not fully describe the hole’s position relative to the joint’s base length. A detailed illustration of these geometric relationships is provided in Appendix C.

#### Design space coverage of L-joint through hybrid CCD-BBD dataset

The hybrid CCD-BBD methodology generated 125 simulations (49 from BBD and 77 from CCD with a shared central point), ensuring five levels per parameter (Table 10) while maintaining geometric constraints outlined in Section "Constraints and criteria for dataset generation". The dataset was subsequently transformed into dimensionless ratios, as detailed in Section "Transforming design parameters into dimensional ratios". To illustrate dataset distribution, a 3D scatter plot is provided in Appendix D, showcasing the design space representation across six design variables. Some points exhibit up to eight overlapping runs, while others display four runs at the same coordinate due to the factorial nature of the design. The range of key ratios (R/L, E/L, W/D, and E/D) is detailed in Appendix E, ensuring comprehensive coverage of the design space.

**Table 10:** Combined parameter levels for CCD and BBD designs.

	CCD					BBD		
	− 2	− 1	0	1	2	− 1	0	1
R (mm)	6.75	14.00	19.00	24.00	31.25	14.00	19.00	24.00
L (mm)	99.63	125.00	142.50	160.00	185.37	125.00	142.50	160.00
W (mm)	33.26	55.00	70.00	85.00	106.74	55.00	70.00	85.00
E (mm)	42.75	50.00	55.00	60.00	67.25	50.00	55.00	60.00
***φ***	1.00	1.09	1.15	1.21	1.30	1.09	1.15	1.21
T (N m)	0.00	10.36	17.50	24.64	35.00	10.36	17.50	24.64

### Feature selection and analysis

#### Identification of significant features using LASSO

Least Absolute Shrinkage and Selection Operator (LASSO) regression was employed to identify significant features by shrinking less relevant coefficients to zero, enhancing both interpretability and model accuracy. Polynomial degrees ranging from 1 to 3 were evaluated using LassoCV with fivefold cross-validation, and the dataset was split into 70% training and 30% testing after standardization. Model performance was evaluated using Root Mean Square Error (RMSE) and the Coefficient of Determination (R^2^).

The quadratic model (degree 2) exhibited the best balance between complexity and predictive accuracy, with the lowest RMSE and highest R^2^, as shown in Table 11. Assessments using residual plots (Appendix F, Fig. F1–F3) confirmed this finding. The quadratic model demonstrated a random distribution of residuals, suggesting a strong fit without overfitting, unlike the cubic model, which exhibited greater residual spread. The significant features identified by the quadratic LASSO model are presented in Appendix F, Table F1.

**Table 11:** Comparison of RMSE and R^2^ score for polynomial models based on test data.

Polynomial degree	Root mean squared error (RMSE)	Predicted R^2^ score
1	0.0516	0.9844
2	0.0360	0.9924
3	0.0698	0.9714

#### Identification of significant features using ANOVA

Following the identification of significant features through the LASSO quadratic model, an Analysis of Variance (ANOVA) was conducted to assess the influence of these features on the response statistically. The ANOVA analysis confirmed the importance of key terms, with E/L, W/D, and E/D emerging as significant contributors. The full list of statistically significant terms, ranked by F-value, is presented in Appendix G, Table G1.

#### Comparison of LASSO and ANOVA for PySR model development

Both LASSO and ANOVA consistently identified the dimensionless ratios E/L, W/D, and E/D as primary contributors to the response, with E/L demonstrating the strongest influence, including non-linear effects and interactions. LASSO highlighted the importance of interaction terms such as (E/L)(E/D) and (E/L)(W/D), while ANOVA emphasized the significance of these features as standalone terms.

For R/L, both methods agreed that its quadratic effect was negligible, though ANOVA identified it as statistically significant on its own. LASSO, on the other hand, captured its relevance through interactions such as (R/L)(Tn) and (R/L)(E/D). Similarly, for the clearance ratio 1/φ and torque Tn, LASSO minimized their individual contributions but retained their effects through interactions, indicating their relevance primarily in combination with other features.

Overall, the agreement between LASSO and ANOVA confirmed the central role of the selected dimensionless ratios, supporting their inclusion in the symbolic regression analysis using PySR.

### Machine learning models development

#### Model selection rationale

The development and comparison of machine learning (ML) models in this study serve two primary objectives. First, they assess whether the hybrid dataset generated using Central Composite Design (CCD) and Box-Behnken Design (BBD) sufficiently captures the design space to enable the discovery of generalizable expressions. Successful generalization by ML models would validate the dataset’s robustness and justify the application of symbolic regression using PySR. Second, the best-performing ML models establish benchmark metrics for predictive accuracy and generalization, which any PySR-derived expression must match or exceed.

The selected models, LASSO, Ridge, Huber, and Support Vector Regression (SVR), employ different regularization techniques to prevent overfitting and maintain generalization across the design space. LASSO regression uses L1 regularization, driving some coefficients to zero for improved interpretability and computational efficiency. Ridge regression applies L2 regularization, penalizing squared coefficients while retaining all features to capture subtle interactions. Huber regression balances L1 and L2 regularization, offering robustness to outliers by minimizing their influence on model predictions. SVR employs an epsilon-insensitive loss function, focusing on global trends while disregarding small errors within a defined margin.

Advanced models, such as deep learning and ensemble methods, were excluded since the selected ML models demonstrated strong predictive performance without adding unnecessary computational complexity, as elaborated in the subsequent sections. Furthermore, the insights gained from these models guided the selection of the loss function for PySR, ensuring the derivation of interpretable, accurate, and generalizable expressions.

#### Selected validation cases for the prediction of unseen data

To assess the models’ ability to generalize beyond the training data, eight additional cases were introduced, covering a broader range of geometric configurations not included in the main dataset. These cases feature three distinct bolt sizes (M5, M8, and M10) as summarized in Table 12, adding complexity by varying dimensionless ratios such as E/L and E/D. While the primary dataset was based on an M8 bolt size, the inclusion of M5 and M10 configurations ensures testing across a wider design space. Some cases, like Case 1, were experimentally validated, enhancing the reliability of the predictions. Cases 7 and 8 were selected to evaluate model sensitivity, as they share nearly identical geometric configurations but differ in bolt sizes and tightening torques. The distribution of these validation cases, distinct from the training data, is illustrated in a 3D scatter plot and a corresponding 2D projection (Appendix H). These visualizations highlight how the selected cases extend beyond the design space of the main dataset, ensuring diverse geometric coverage.

**Table 12:** Summary of selected cases for model prediction with corresponding bolt sizes.

	Bolt size	R/L	W/D	E/D	E/L	1/φ	Tn	FEA damage initiation load (kN)
Case 1	M8	0.12	8.375	8.00	0.43	0.89	1.00	2.38
Case 2	M10	0.11	7.00	5.50	0.44	0.83	0.60	3.29
Case 3	M5	0.17	9.00	12.00	0.50	0.89	0.70	2.51
Case 4	M5	0.19	11.00	10.00	0.40	0.86	0.80	1.95
Case 5	M10	0.09	8.50	6.00	0.38	0.91	1.00	2.68
Case 6	M8	0.19	8.75	3.75	0.30	0.87	0.61	2.83
Case 7	M10	0.22	12.91	5.53	0.55	0.91	0.67	6.30
Case 8	M8	0.22	13.34	5.34	0.55	0.77	0.23	5.51

#### Models comparison and performance evaluation

All models followed a consistent methodology, including data preprocessing, training-validation splits, and hyperparameter tuning, to ensure fair comparisons. The analysis evaluated model performance across two data splits (70–30 and 90–10) to identify the most robust model for comparison with PySR. The results are summarized in Appendix I (Tables I1–I3). The RMSE and R^2^, shown in Appendix I, Table I1, indicate consistently high performance across all models, with minor RMSE differences and R^2^ values close to 0.99 for both splits. However, the percentage error on validation cases provided a clearer measure of generalization ability (Appendix I, Tables I2–I3):

Huber Regression consistently outperformed other models with the lowest average errors in both splits (approximately 5.96%), without exceeding a 10% error threshold in any case. Its robustness in handling high-variation cases, such as Case 3, highlights its reliability.

SVR delivered strong overall performance but showed inconsistencies, particularly in Case 3 and Case 6 under the 70–30 split, where errors exceeded 10%. Some improvement was noted in the 90–10 split, though errors remained above 10% for certain cases.

Ridge Regression achieved the lowest RMSE and highest R^2^ values but struggled with certain cases, particularly Case 3 and Case 6. Its high sensitivity to challenging configurations reduced its overall reliability.

LASSO Regression performed the least reliably, with the highest average errors in both splits (10.39% for 70–30 and 10.49% for 90–10). It consistently underperformed in complex cases, particularly Case 3, where errors exceeded 30%.

#### Baseline model for comparison and insights for PySR

Huber regression was selected as the baseline model due to its strong balance between RMSE, R^2^, and percentage error. It consistently demonstrated superior generalization, robust handling of challenging configurations, and stable performance across both data splits. This makes it the most reliable model for comparison with the PySR symbolic regression model in subsequent analyses. The standardized equation for the Huber model is presented in Eq. (5).

The consistently high R^2^ values and low RMSE across all models confirm that the dataset effectively captures key trends and interactions within the design space. The strong performance of Huber Regression on unseen cases, particularly in challenging scenarios like Case 3, further validates the dataset’s ability to generalize beyond the sampled points. These findings suggest that the systematic sampling provided by CCD and BBD enhances the potential for PySR to derive interpretable expressions that accurately represent the underlying behavior of the design space.

5 $$\begin{aligned} Huber\,Damage\,Initian\,Load \left( {kN} \right) & = 2.3851 - 0.1231\left( \frac{R}{L} \right) + 0.0164\left( \frac{W}{D} \right) - 0.1309\left( \frac{E}{D} \right) \\ & \quad + 0.1065\left( \frac{E}{L} \right) + 0.303\left( {\frac{1}{\varphi }} \right) + 0.0714 \left( {Tn} \right) + 0.0313\left( \frac{R}{L} \right)^{2} \\ & \quad + 0.0738\left( \frac{R}{L} \right)\left( \frac{W}{D} \right) - 0.0364\left( \frac{R}{L} \right)\left( \frac{E}{D} \right) + 0.1586\left( \frac{R}{L} \right)\left( \frac{E}{L} \right) \\ & \quad - 0.0054\left( \frac{R}{L} \right)\left( {\frac{1}{\varphi }} \right) - 0.0275\left( \frac{R}{L} \right)\left( {Tn} \right) - 0.1365\left( \frac{W}{D} \right)^{2} \\ & \quad + - 0.0239\left( \frac{W}{D} \right)\left( \frac{E}{D} \right) + 0.2424\left( \frac{W}{D} \right)\left( \frac{E}{L} \right) + 0.0547 \left( \frac{W}{D} \right)\left( {\frac{1}{\varphi }} \right) \\ & \quad + 0.0541\left( \frac{W}{D} \right)\left( {Tn} \right) + 0.2114\left( \frac{E}{D} \right)^{2} - 0.5041\left( \frac{E}{D} \right)\left( \frac{E}{L} \right) \\ & \quad - 0.0234\left( \frac{E}{D} \right)\left( {\frac{1}{\varphi }} \right) - 0.0598\left( \frac{E}{D} \right)\left( {Tn} \right) + 0.5164\left( \frac{E}{L} \right)^{2} \\ & \quad + 0.0296\left( \frac{E}{L} \right)\left( {\frac{1}{\varphi }} \right) + 0.0502\left( \frac{E}{L} \right)\left( {Tn} \right) - 0.2675\left( {\frac{1}{\varphi }} \right)^{2} \\ & \quad - 0.0552\left( {\frac{1}{\varphi }} \right)\left( { Tn} \right) - 0.0171\left( {Tn} \right) \\ \end{aligned}$$

### PySR modeling and evaluation

The tuning of PySR parameters followed an iterative process aimed at balancing interpretability and predictive accuracy. The focus was on deriving compact, physically meaningful expressions while ensuring strong model performance. Key features identified by LASSO and ANOVA were prioritized, and model performance was evaluated using RMSE and R^2^ metrics. Validated expressions were compared against the Huber regression baseline using an unseen dataset and assessment through residual plots.

The operator set for PySR included exponential, logarithmic, trigonometric, hyperbolic functions, and square roots, with polynomial terms implicitly generated by PySR. To improve interpretability, constraints were applied to prevent nested functions such as sin(*e*^*x*^) or $e^{{e^{x} }}$. Fundamental binary operations such as addition, subtraction, multiplication, and division formed the core of candidate expressions. The population size was increased progressively from 100 to 1200, while maintaining the default 31 islands to ensure solution diversity. The iteration count scaled similarly, and the maxsize parameter, controlling equation complexity, was adjusted from 10 to 23 to ensure the inclusion of essential dimensionless ratios identified by LASSO and ANOVA.

Although the Huber loss function initially showed promise, its complexity due to the need for a transition parameter led to its replacement with the log-cosh loss function. The latter offered similar robustness without additional tuning, making it better suited for symbolic regression while maintaining predictive accuracy similar to Huber^61^.

The computational process, involving 180 trials, was resource-intensive, utilizing parallel execution on high-performance platforms: Rescale Cloud Computing Service (Intel Xeon, 3.5 GHz, 32 cores) and Google Colab (Intel Xeon, 2.20 GHz, 2 virtual cores). The final symbolic regression expression, presented in Eq. (6), was derived after extensive exploration. Detailed configuration parameters are available in Appendix J, Table J1.

6 $$\begin{gathered} {\text{Damage}}\,{\text{Initiation}}\,{\text{Load}}\,\left( {{\text{kN}}} \right) = a_{1} \left( {e^{{c_{1} \frac{E}{L}}} } \right)\left( {e^{{\frac{{c_{2} }}{{\varphi \left( {E/D} \right)}}}} } \right)\left( {e^{{\frac{{c_{3} }}{{\varphi \left( {W/D} \right)}}}} } \right)\left( {e^{{c_{4} \frac{E/D}{{W/D}}}} } \right) + a_{2} \left( {\frac{R}{L}T_{n} } \right) \hfill \\ where \left\{ {\begin{array}{*{20}l} {a_{1} = a_{2} = 0.4686 } \hfill \\ {c_{1} = c_{2} = 3.9387 } \hfill \\ {c_{3} = - 1 } \hfill \\ {c_{4} = - 0.4244 } \hfill \\ \end{array} } \right. \hfill \\ \end{gathered}$$

## Results

### Performance comparison between PySR and huber regression

#### Performance metrics on main dataset

Both the PySR and Huber Regression models demonstrated strong predictive performance on the main dataset, as shown in Appendix K, Table K1. The Huber Regression achieved an RMSE of 0.0679 and an R^2^ value of 0.9894, while PySR slightly outperformed it with an RMSE of 0.0630 and an R^2^ of 0.9908. These results confirm both models’ ability to capture the underlying relationships between input variables and the damage initiation load.

#### Prediction accuracy on validation cases

The predictive performance was further validated using eight independent cases (results in Appendix K, Table K2). The PySR model achieved a lower average error (4.66%) compared to Huber Regression (5.96%), particularly excelling in challenging configurations such as Cases 3 and 6, where it produced lower error rates.

#### Prediction accuracy on validation cases

The residual plots comparisons of the two models further illustrate their predictive accuracy. As shown in Appendix K, Figures K1–K3, both models exhibit strong alignment with the ideal prediction line in the Predicted vs. Actual plots, confirming high accuracy across the dataset. However, the PySR model demonstrated better consistency in residual distribution, with residuals more evenly distributed around zero (Fig. K2). Additionally, the residual histograms reveal that the PySR model’s residuals were more symmetrically distributed and narrower compared to those of the Huber Regression (Fig. K3), indicating a slight edge in consistency and generalization.

### Physical interpretability of PySR-derived equations

The PySR-derived equation (Eq. 6) offers a simplified, interpretable representation of the factors influencing damage initiation, providing clearer physical insights than the Huber Regression model (Eq. 5). While the Huber model achieves accurate predictions using a complex high-order polynomial with numerous coefficients and interactions, its complexity obscures the underlying physical relationships between variables. In contrast, PySR balances predictive accuracy with interpretability, making it a more effective tool for understanding and guiding design decisions. Each term in the PySR equation reflects a distinct geometric or physical property of the bolted joint:

- The constants a_1_ and a_2_ act as scaling factors, adjusting the contributions of the exponential and linear terms to ensure appropriate weighting of geometric and mechanical influences.
- The exponential terms capture key geometric effects, isolating distinct relationships that influence damage initiation.
  - $\left( {e^{{c_{1} \frac{E}{L}}} } \right)$, quantifies the effect of hole position relative to the base length,
  - $\left( {e^{{\frac{{c_{2} }}{{\varphi \left( {E/D} \right)}}}} } \right)$ and $\left( {e^{{\frac{{c_{3} }}{{\varphi \left( {W/D} \right)}}}} } \right)$ describes the influence of hole size relative to edge and width distance respectively, modulated by the hole size multiplier (***φ***).
  - $\left( {e^{{c_{4} \frac{E/D}{{W/D}}}} } \right)$ captures the interaction between edge distance and plate width. When rewritten as $\left( {e^{{c_{4} \frac{E}{W}}} } \right)$, the latter term suggests an additional aspect ratio effect associated with the prying action area.
- The linear term reflects an interaction between tightening torque (Tn) and the fillet-to-length ratio (R/L), capturing the impact of stress distribution around the hole and its proximity to the fillet.

Notably, PySR exhibits symmetry in its treatment of W/D and E/D. Despite lacking predefined knowledge of their physical significance, the model identifies their importance and represents them in a balanced manner. Additionally, the hole size multiplier (***φ***) appears in key interactions, reinforcing its role in modifying hole-dependent dimensionless ratios.

### Sensitivity analysis through symbolic derivatives

To complement the interpretability of the symbolic regression model, a first-order sensitivity analysis was conducted by analytically computing the partial derivatives of the PySR-derived expression with respect to each of its input variables. To streamline the presentation of the partial derivatives, Eq. (7) introduces the substitution for the common exponential term that appears throughout the symbolic regression model. The resulting partial derivatives are summarized in Table 13.

7 $$\Psi = e^{{c_{1} \frac{E}{L} + \frac{{c_{2} }}{{\varphi \left( {E/D} \right)}} + \frac{{c_{3} }}{{\varphi \left( {W/D} \right)}} + c_{4} \frac{E/D}{{W/D}}}}$$

**Table 13:** First-order sensitivity analysis of the symbolic regression model.

Variable	Partial derivative expression
E/L	$a_{1} c_{1} \Psi$
E/D	$a_{1} \left[ { - \frac{{c_{2} }}{{\varphi \left( {E/D} \right)^{2} }} + \frac{{c_{4} }}{{\left( {W/D} \right)}}} \right]\Psi$
W/D	$a_{1} \left[ { - \frac{{c_{3} }}{{\varphi \left( {W/D} \right)^{2} }} - \frac{{c_{4} \left( {E/D} \right)}}{{\left( {W/D} \right)^{2} }}} \right]\Psi$
R/L	$a_{2} T_{n}$
***φ***	$a_{1} \left( { - \frac{{c_{2} }}{{\varphi^{2} \left( {E/D} \right)}} - \frac{{c_{3} }}{{\varphi^{2} \left( {W/D} \right)}}} \right)\Psi$
Tn	$a_{2} \left( {R/L} \right)$

The partial derivative with respect to E/L shows a direct and positive contribution to the predicted damage initiation load, as both a_1_ and c_1_ are positive in Eq. (6). Since this term appears as a multiplicative factor within the exponential function Ψ, increasing the edge distance relative to the plate length leads to a consistent exponential rise in load capacity.

In the case of E/D, the derivative consists of two competing components: an inverse-square term arising from the clearance-sensitive expression and a linear term resulting from the prying-effect interaction with W/D. Given the coefficient values in Eq. (6), the combined influence is nonlinear and predominantly negative.

The derivative with respect to W/D similarly includes terms from both the clearance-related expression and the interaction with E/D. The overall influence is positive considering the coefficients in Eq. (6) , the presence of W/D in squared denominators introduces diminishing returns. That is, increasing plate width enhances load capacity, but the benefit tapers off as width continues to grow. This behavior aligns with physical expectations, where wider plates distribute stress more effectively up to a certain point.

The partial derivative with respect to the clearance multiplier ***φ*** is nonlinear and primarily negative, indicating that increases in clearance tend to reduce the predicted damage initiation load. The derivative includes two terms: the first, proportional to − c_2_, is always negative and dominates when E/D is not large. The second term, involving − c3, is positive and introduces a moderating effect, especially when W/D is relatively small. The presence of ***φ***^2^ in the denominator of both terms results in diminishing returns that is the sensitivity is highest at low clearance levels and gradually decreases as ***φ*** increases. The effect is also geometry-dependent, governed by the interaction between clearance and the ratios E/D and W/D.

The partial derivatives with respect to the normalized torque Tn​ and R/L exhibit a simple linear relationship and mutual dependency. Specifically, the derivative with respect to Tn​ is directly proportional to R/L, and vice versa. This implies that the beneficial effect of torque on damage initiation load is conditional on having sufficient fillet radius, and conversely, the contribution of the fillet radius becomes significant primarily under applied torque.

## Discussion

### Advantages and applicability of the proposed PySR equation

#### Addressing gaps in design standards through realistic modeling

This study addresses critical limitations outlined by EUROCOMP for FRP bolted connections. The inclusion of factors such as hole clearance enhances the physical realism of the model. This leads to more accurate predictions of damage initiation loads, providing engineers with a tool that reflects real-world joint behavior more reliably.

#### Enhanced design optimization enabled by PySR interpretability

The PySR-derived equation, due to its concise and interpretable mathematical form, allows engineers to optimize joint configurations without relying extensively on computational simulations or experimental testing. The PySR equation not only improves predictive accuracy but also enhances physical interpretability through exponential and linear terms which help identify critical design factors influencing damage initiation, enabling more informed decision-making in practical applications.

#### Scalability across different bolt sizes

Validation cases presented in Section "Selected validation cases for the prediction of unseen data" demonstrate the model’s ability to accurately predict damage initiation loads across varying bolt sizes (M5, M8, M10). This scalability ensures its applicability to a wide range of practical industrial configurations.

#### Scalability Across a Single Row of Equally-Spaced Bolts

The model was also validated for L-joints with equally spaced rows of bolts through finite element simulations incorporating symmetrical boundary conditions to represent the repetitive behavior of unit cells. Ten cases, covering extreme and intermediate geometric configurations (detailed in Appendix L), revealed that the presence of multiple bolts slightly increases the damage initiation load. This confirms the equation’s ability to handle multi-bolt configurations in industrial scenarios.

#### Validation across different stacking sequences

The model was further validated for alternative stacking sequences, such as [45/0/45/0], where it maintained a high predictive accuracy (RMSE = 0.06855, R^2^ = 0.9906). These results, detailed in Appendix M, indicate the flexibility of the PySR-derived equation in capturing the effects of various composite laminate configurations by determining the new coefficient values through fitting (a_1_, a_2_, c_1_, c_2_, c_3_, and c_4_).

### Current limitations and future work

In this study, the current PySR equation does not explicitly capture the effect of thickness on the damage initiation load, as its influence is embedded within the existing coefficients. To address this, future work could introduce dimensionless ratios such as thickness-to-width (t/W) or thickness-to-diameter (t/D) to provide normalized measures of thickness. However, incorporating thickness would require determining whether it should be treated as a standalone term, an interaction effect, or integrated into the model coefficients. Another limitation is that the equation’s applicability is restricted to cases with an equally distributed row of bolts.

## Conclusions

This study presents a comprehensive framework integrating experimental testing, finite element modeling (FEM), and symbolic regression (PySR) to predict damage initiation loads in hybrid GFRP-steel L-joint bolted connections. Unlike traditional machine learning models, the PySR-derived equation offers both high predictive accuracy and clear physical interpretability, providing engineers with an effective tool for optimizing joint performance.

Key contributions of this research include addressing limitations in existing design standards, particularly those outlined by EUROCOMP, by incorporating real-world parameters such as hole clearance and friction effects. The model’s reliance on dimensionless geometric ratios ensures scalability across various bolt sizes and joint configurations. Validation against unseen cases, multi-bolt assemblies, and alternative stacking sequences, further demonstrated the equation’s robustness and flexibility.

The PySR equation not only simplifies complex relationships between geometric and mechanical variables but also enables faster design optimization by reducing the need for extensive simulations and experimental tests. Its interpretability allows for the identification of critical design factors such as edge distance (E/D), plate width (W/D), and tightening torque (Tn), facilitating informed design decisions in industrial applications.

This study offers a scalable, interpretable, and predictive framework for hybrid bolted joints, bridging the gap between theoretical models and real-world engineering applications across industries such as aerospace, automotive, and civil engineering.

## Supplementary Information

Supplementary Information.

## Data Availability

The datasets generated in the current study are available from the corresponding author on request.
