# Supplementary material for: Integrating machine learning and symbolic regression for predicting damage initiation in hybrid FRP bolted connections
Source: Sci Rep. 2025 May 27;15:18564. doi: 10.1038/s41598-025-02390-4 (PMC12117092; doi:10.1038/s41598-025-02390-4)
Supplement: Supplementary file 1 — Supplementary Information. [file 41598_2025_2390_MOESM1_ESM.pdf]

Appendix A. Manufacturing and Visual Inspection of Machined GFRP Specimens

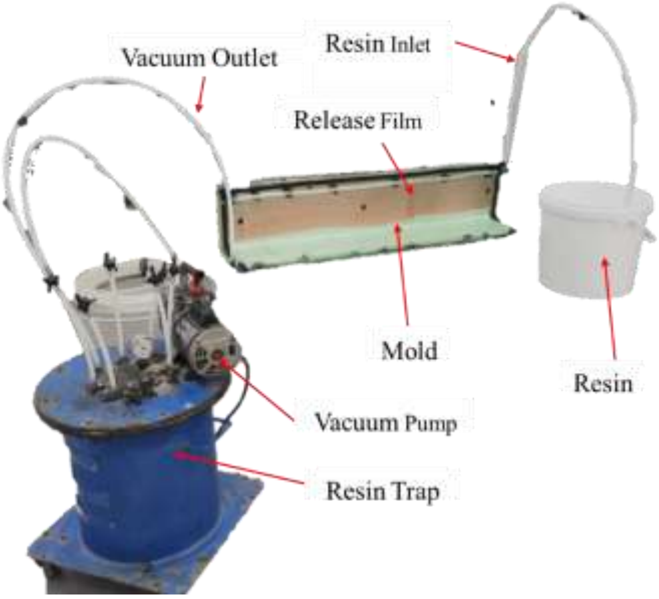

Fig. A1 Vacuum-Assisted Resin Infusion Process for Composite L-Part

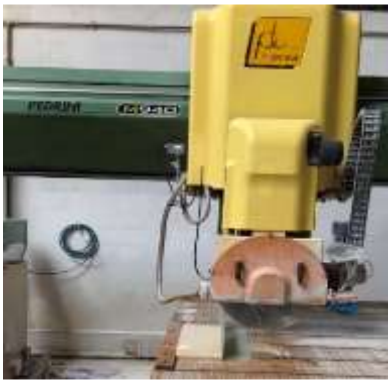

(a) Pedrini M940 CNC

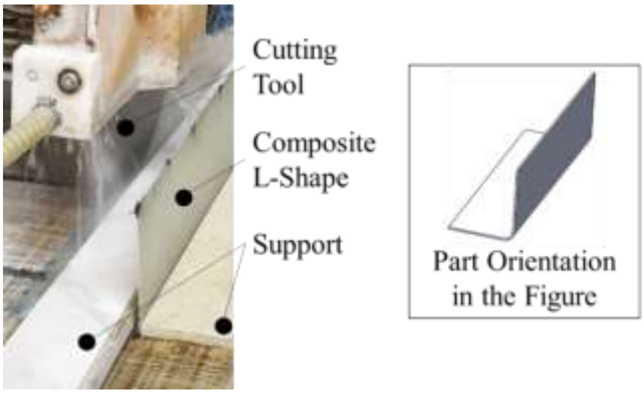

(b) Trimming of the Composite Part Periphery

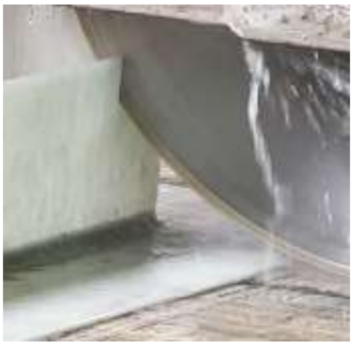

(c) Division of the Composite Part into Samples

Fig. A2 L-Shape Cutting Process on the Pedrini M940 CNC

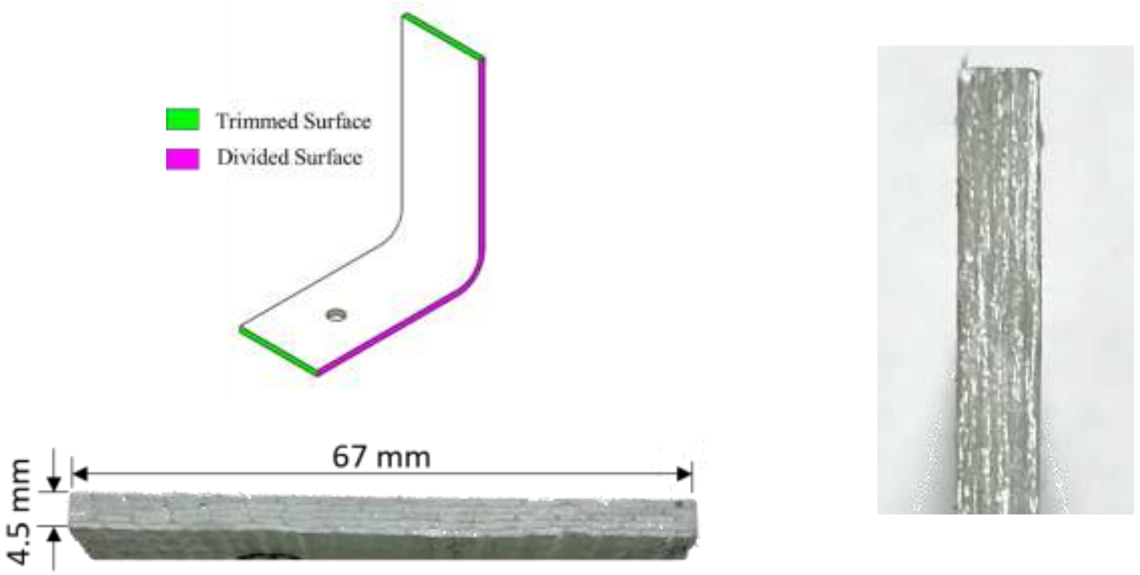

(a) Composite Surface after Trimming (b) Composite Surface after Division

Fig. A3 Visual Inspection After Cutting Process

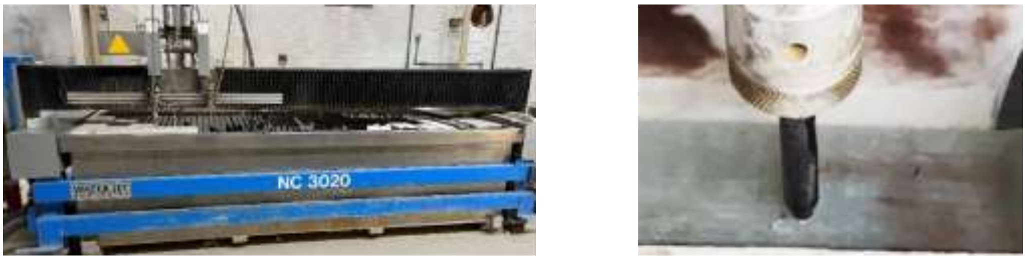

(a) Waterjet Sweden NC 3020 (b) Hole Machining

Fig. A4 Machining Holes with Abrasive Waterjet on the Waterjet Sweden NC 3020 Machine

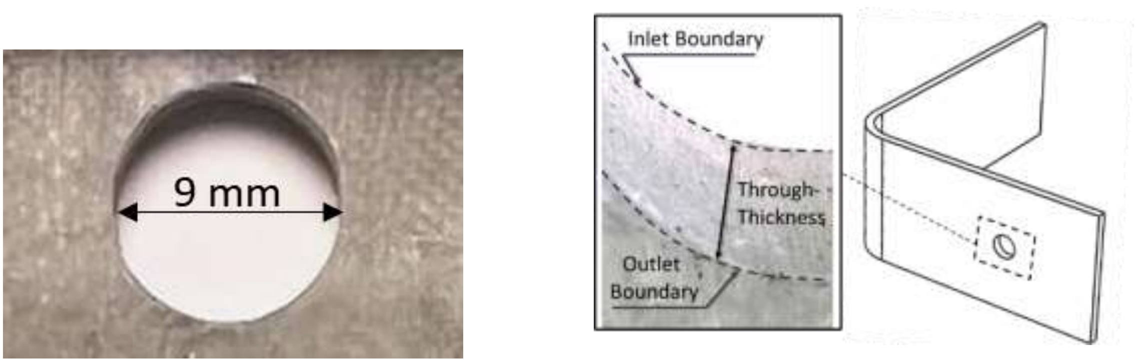

(a) Machined Hole Entry (b) Hole Though-Thickness

Fig. A5 Visual Inspection of the Machined Holes for Absence of Delamination Damage.

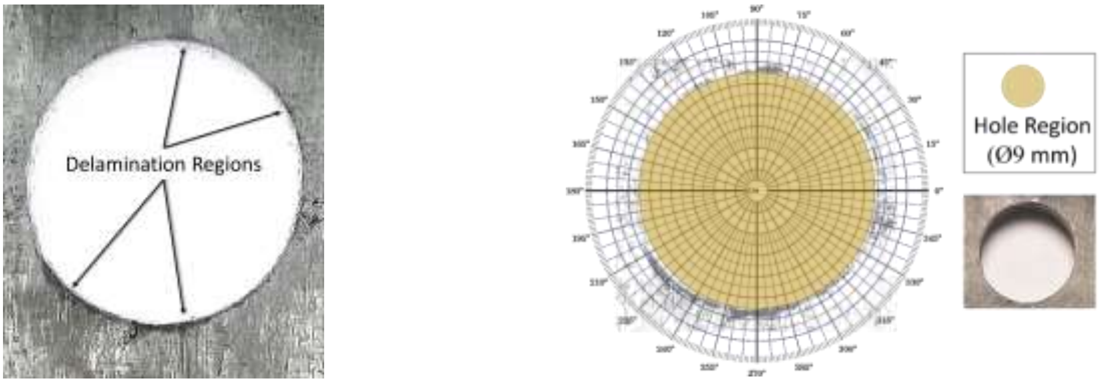

(a) Grey-Scaled Backlight Image (b) Quantification of Damage Extent

Fig. A6 Outcome of Image Processing for Medium-Fit M8 hole.

Appendix B. Visualization of Intralaminar Damage in Composite L-Joint Concurrent with Matrix Tension Failure Initiation

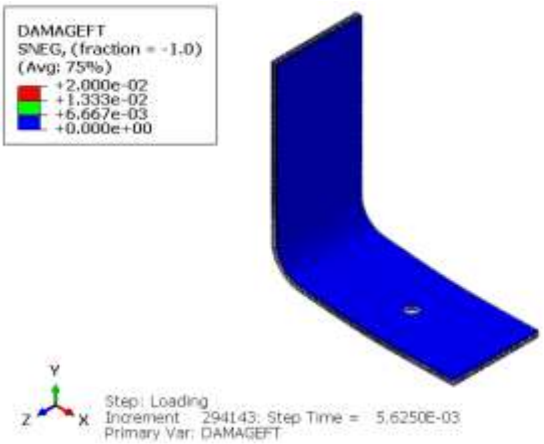

(a) Fiber Tension Failure

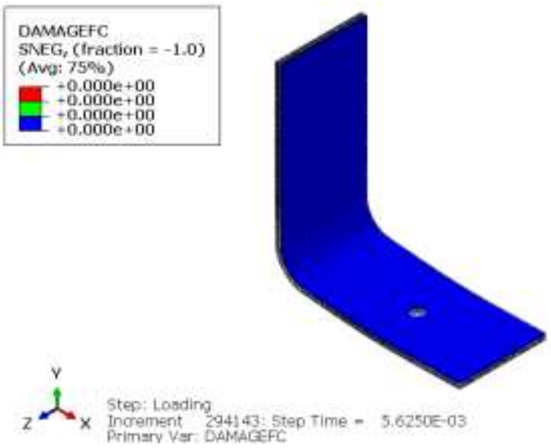

(b) Fiber Compression Failure

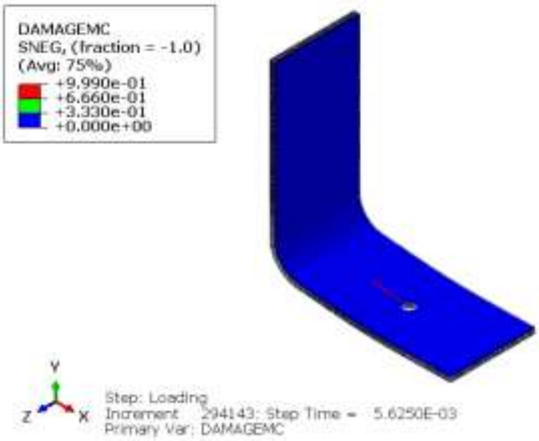

(c) Matrix Compression Failure

Fig. B1 Intralaminar Damage Analysis Concurrent with Matrix Tension Failure Initiation

Appendix C. Necessity of the E/L Ratio for Capturing Geometric Variations in L-Joints

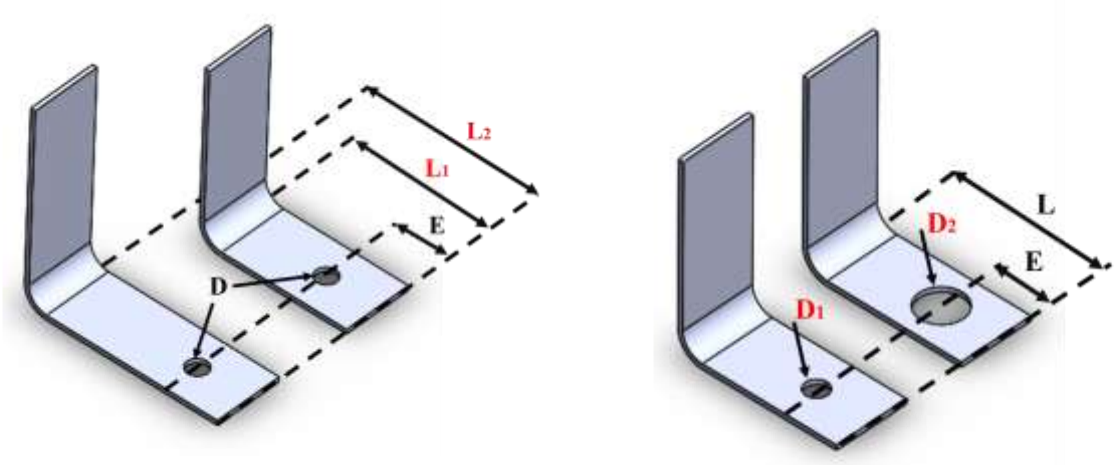

(a) Same E/D but different E/L (b) Same E/L but different E/D

Fig. C1 Illustration of E/L Ratio's Role in Capturing Geometric Variations in L-Joints.

Appendix D. Visualizations of Design Space Coverage

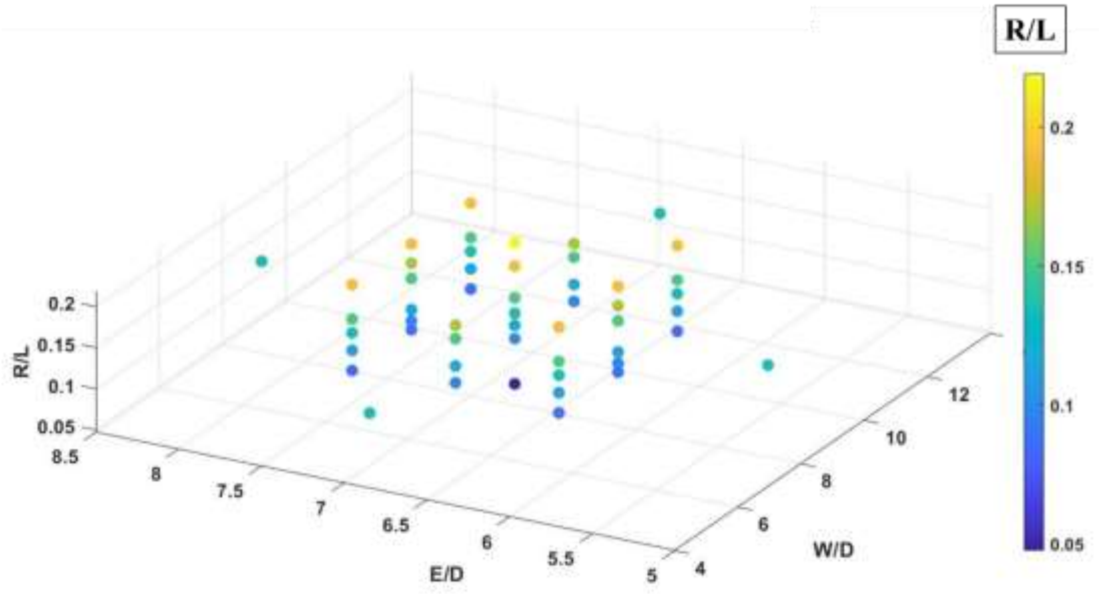

Fig. D1 3D Scatter Plot of W/D, E/D, and R/L, color-mapped based on R/L values

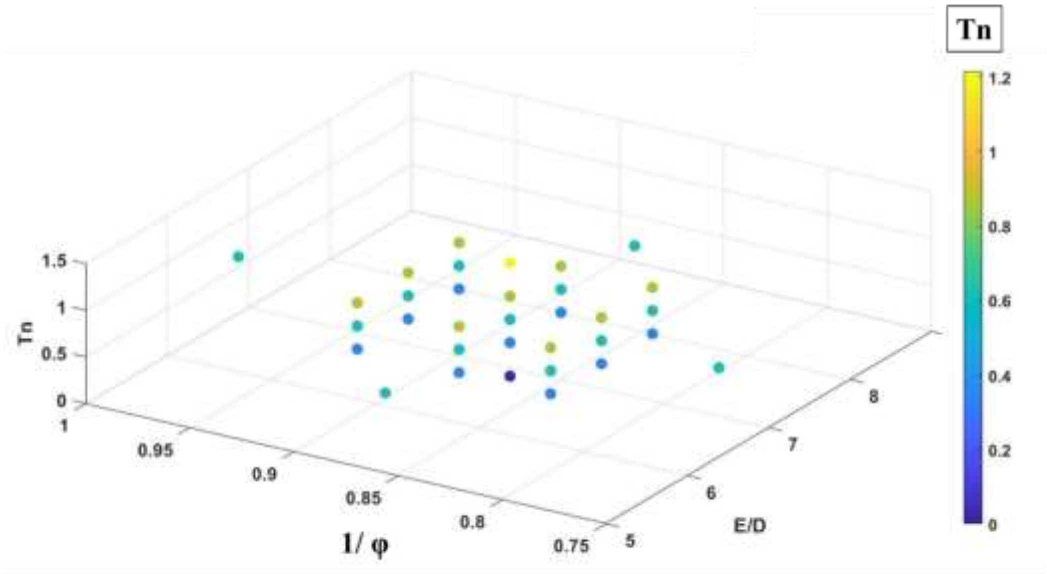

Fig. D2 3D Scatter Plot of E/D, 1/ φ, and Tn color-mapped based on Tn values

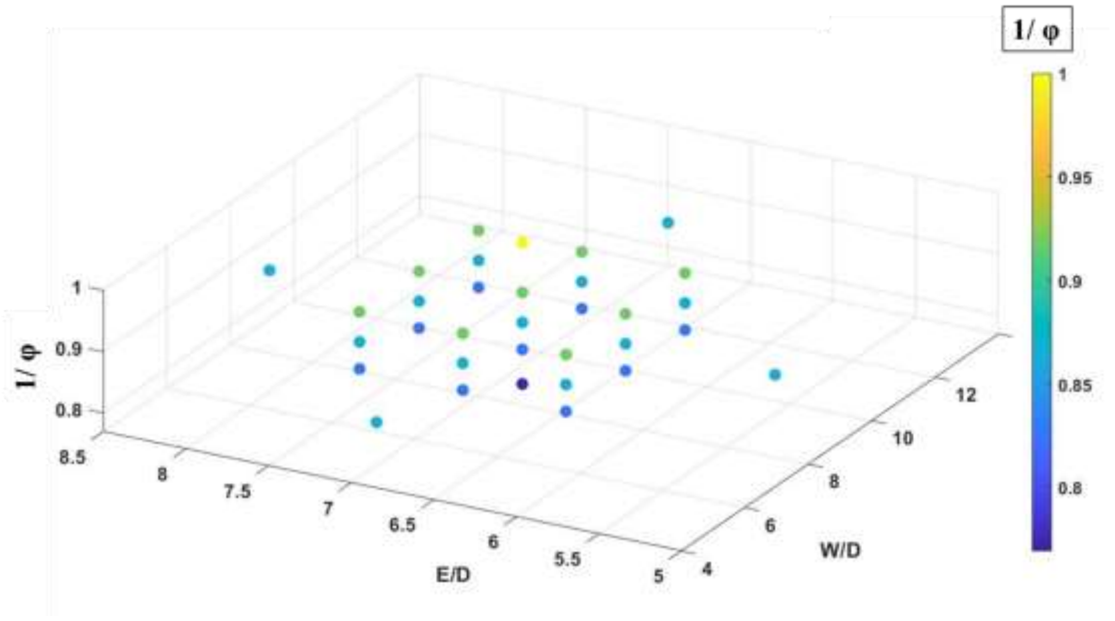

Fig. D3 3D Scatter Plot of W/D, E/D, and 1/ φ, color-mapped based on 1/ φ values

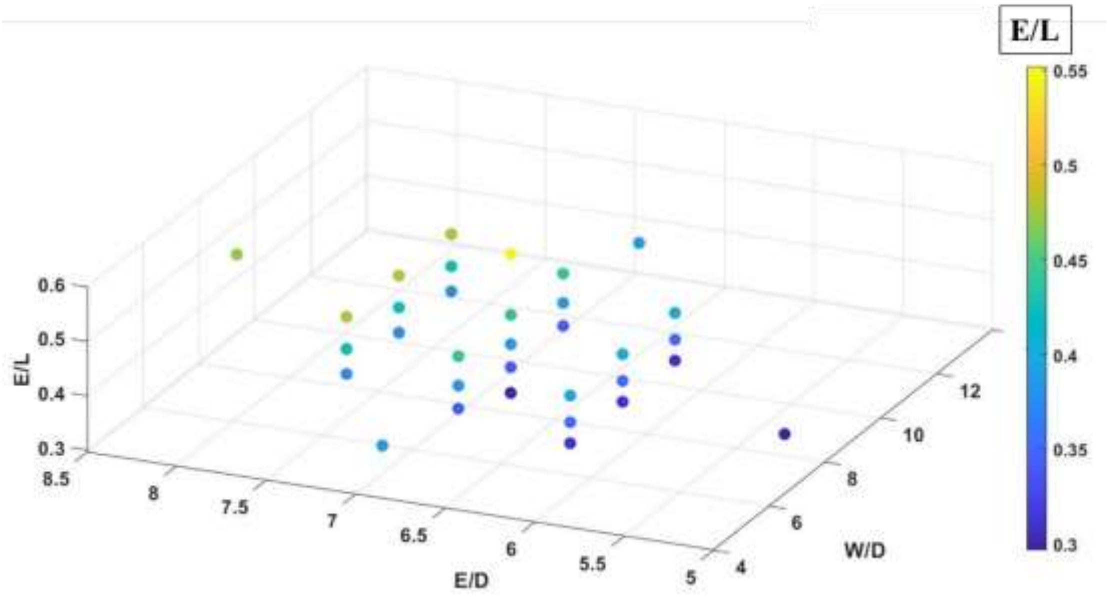

Fig. D4 3D Scatter Plot of W/D, E/D, and E/L, color-mapped based on E/L values

Appendix E. Additional Visualizations of Design Space Coverage

Table E1 Range of Dimensionless Geometric Ratios for L-Joints in the Design Space Study

	**R/L**	**E/L**	**W/D**	**E/D**
**Min**	0.05	0.29	4.15	5.34
	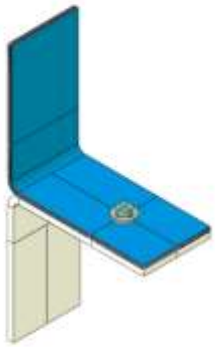	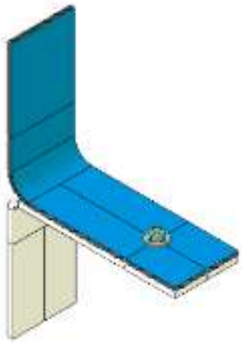	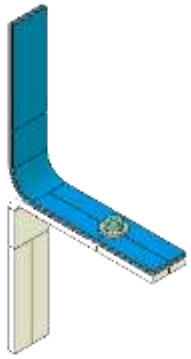	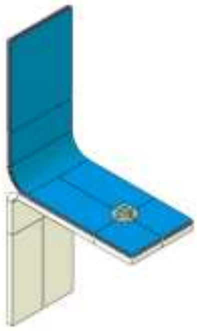
**Max**	0.22	0.55	13.34	8.40
	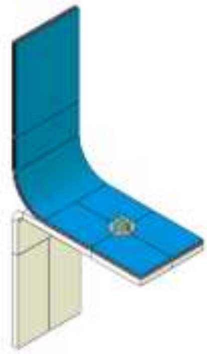	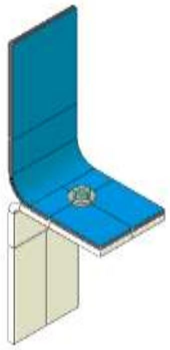	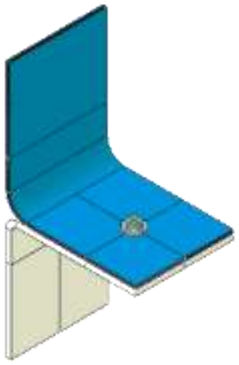	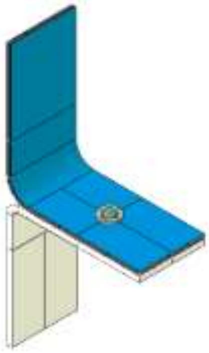

Appendix F. LASSO Regression Results and Model Performance

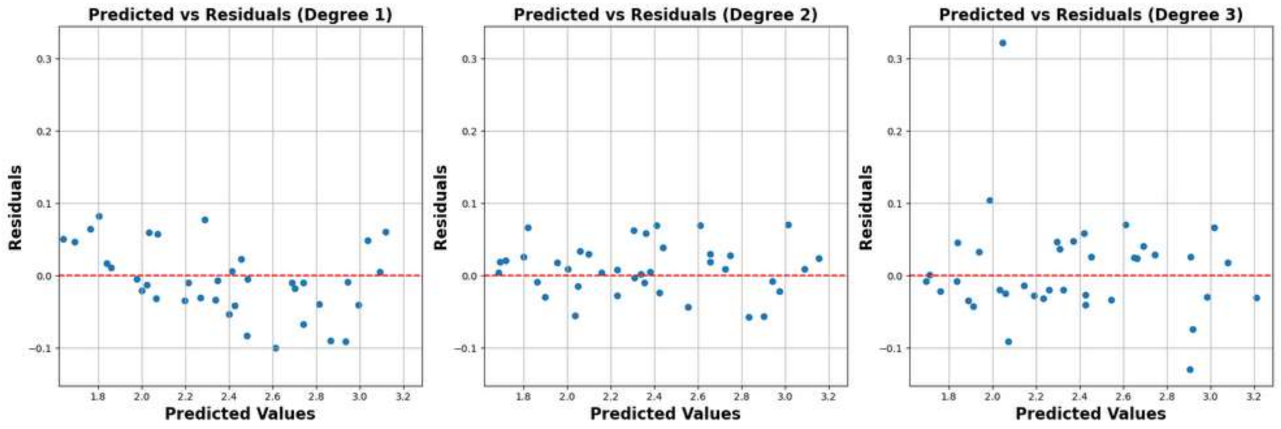

Fig. F1 Comparison of Predicted vs Residuals Plots for Studied Polynomial Degrees

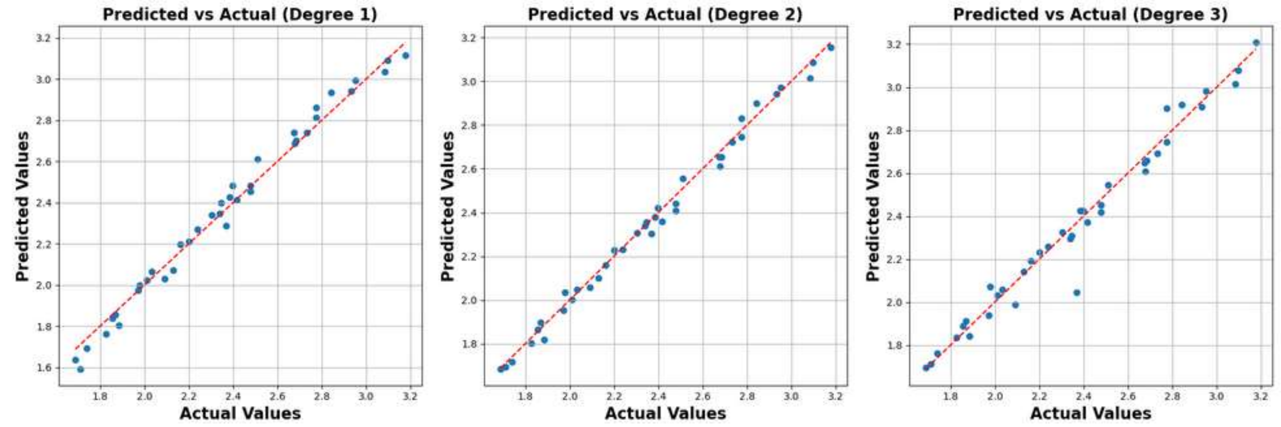

Fig. F2 Comparison of Predicted vs Actual Values Plots for Studied Polynomial Degrees

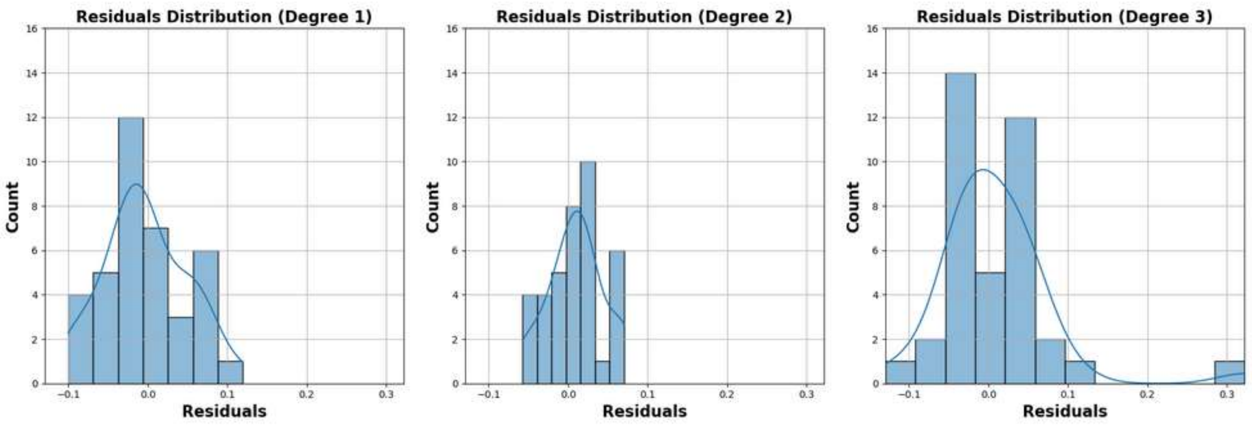

Fig. F3 Comparison of Residuals Distribution Plots for Studied Polynomial Degrees

Table F1 Significant Terms from the LASSO Quadratic Polynomial Model

**Significant Terms**			
**Increase Damage Initiation Load**	**Coefficient**	**Decrease Damage Initiation Load**	**Coefficient**
**$(E/L)^2$**	0.674	**(E/L) (E/D)**	-0.581
**(E/L) (W/D)**	0.226	**$(W/D)^2$**	-0.121
**(E/L) (R/L)**	0.204	**(1/φ) (Tn)**	-0.089
**(W/D)**	0.054	**(R/L)**	-0.083
**(E/L) (Tn)**	0.051	**(R/L) (E/D)**	-0.055
**(W/D) (Tn)**	0.048	**(R/L) (Tn)**	-0.019
**$(E/D)^2$**	0.043	**(R/L) (1/φ)**	-0.006
**(E/L) (1/φ)**	0.039		
**(W/D) (E/D)**	0.039		
**(R/L) (W/D)**	0.038		
**$(Tn)^2$**	0.034		
**(W/D) (1/φ)**	0.034		
**(E/D)**	0.023		
**(1/φ)**	0.019		
**(E/D) (1/φ)**	0.014		

Appendix D. ANOVA Results

Table D1 Significant Terms from ANOVA Analysis for Quadratic Model Ordered by F-value

Term	F-value	P-value
E/L	8279.69	1.76E-96
W/D	3161.43	2.18E-76
E/D	1127.75	1.45E-55
$(E/L)^2$	117.64	1.78E-18
R/L	65.71	1.51E-12
1/φ	66.33	1.25E-12
(E/D)(E/L)	40.17	7.13E-09
(W/D)(E/L)	35.69	3.73E-08
(R/L) (E/L)	13.41	4.10E-04
(W/D)(Tn)	10.88	1.40E-03
$(W/D)^2$	27.76	8.16E-07
(Tn)	20.74	1.52E-05
$(1/\varphi)^2$	8.21	5.10E-03
$(Tn)^2$	7.3	8.10E-03
$(E/D)^2$	4.61	3.42E-02
(R/L)(W/D)	9.22	3.10E-03

Appendix H. Additional Visualizations for Validation Case Distinction

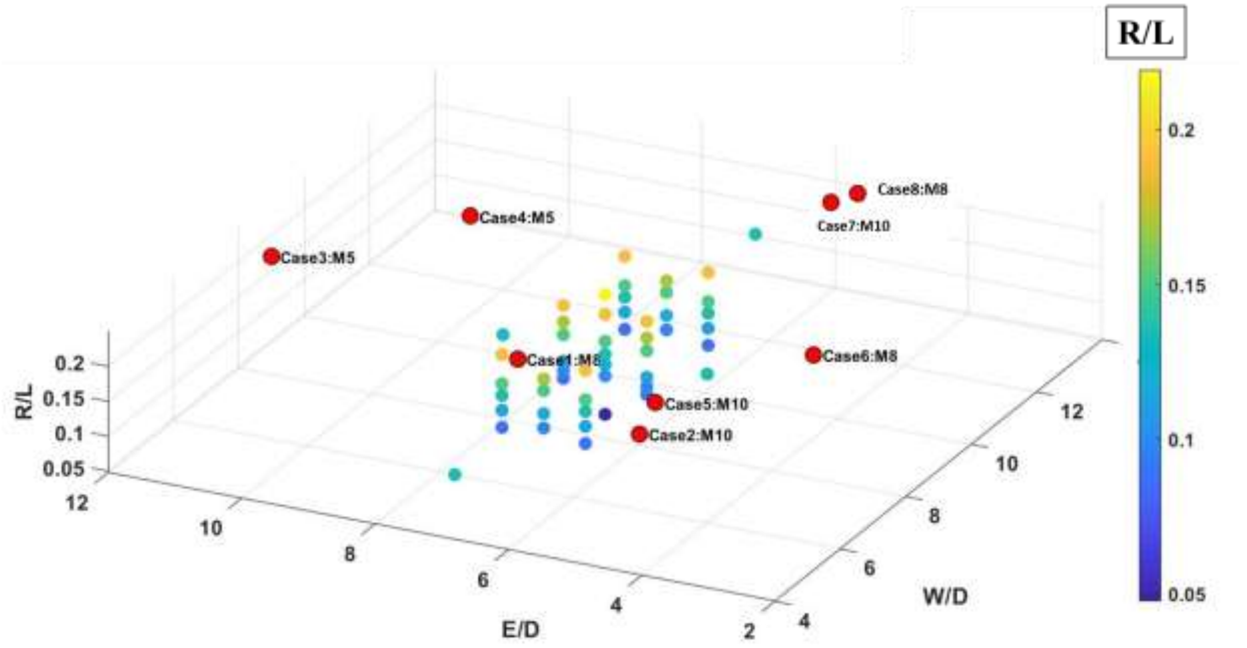

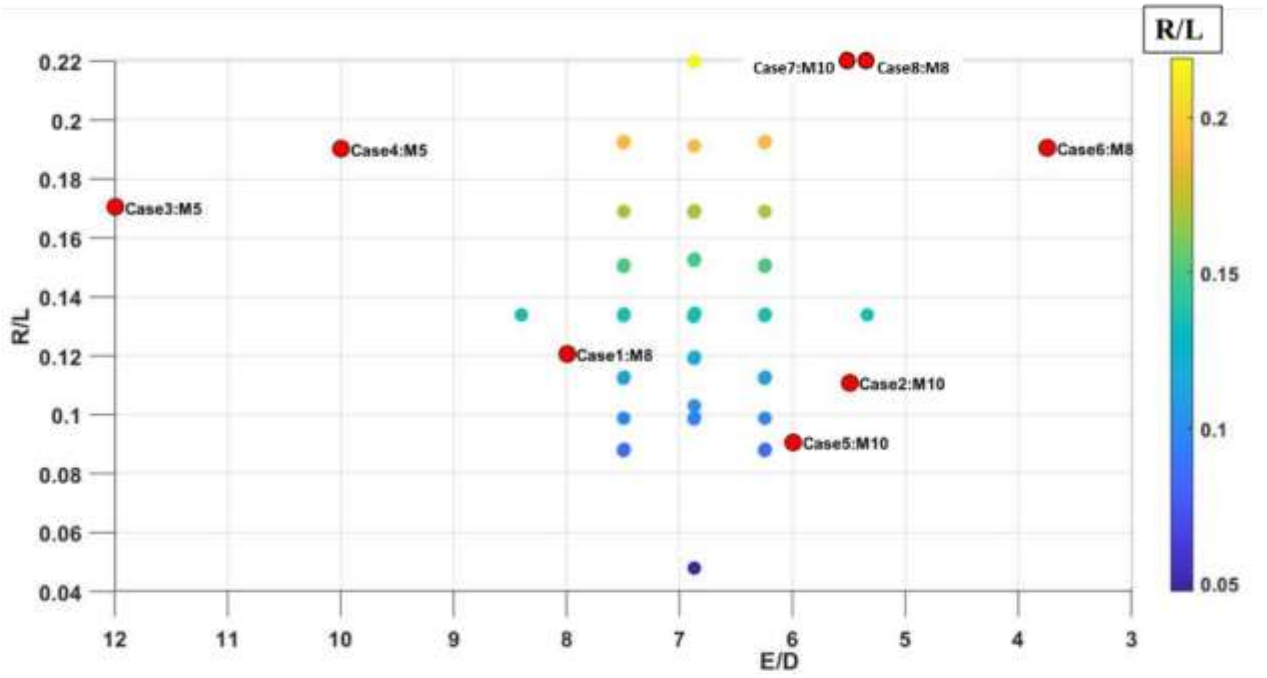

Fig. H1 3D scatter plot of W/D, E/D, and R/L with a 2D projection of E/D versus R/L, color-mapped by R/L, showing validation cases

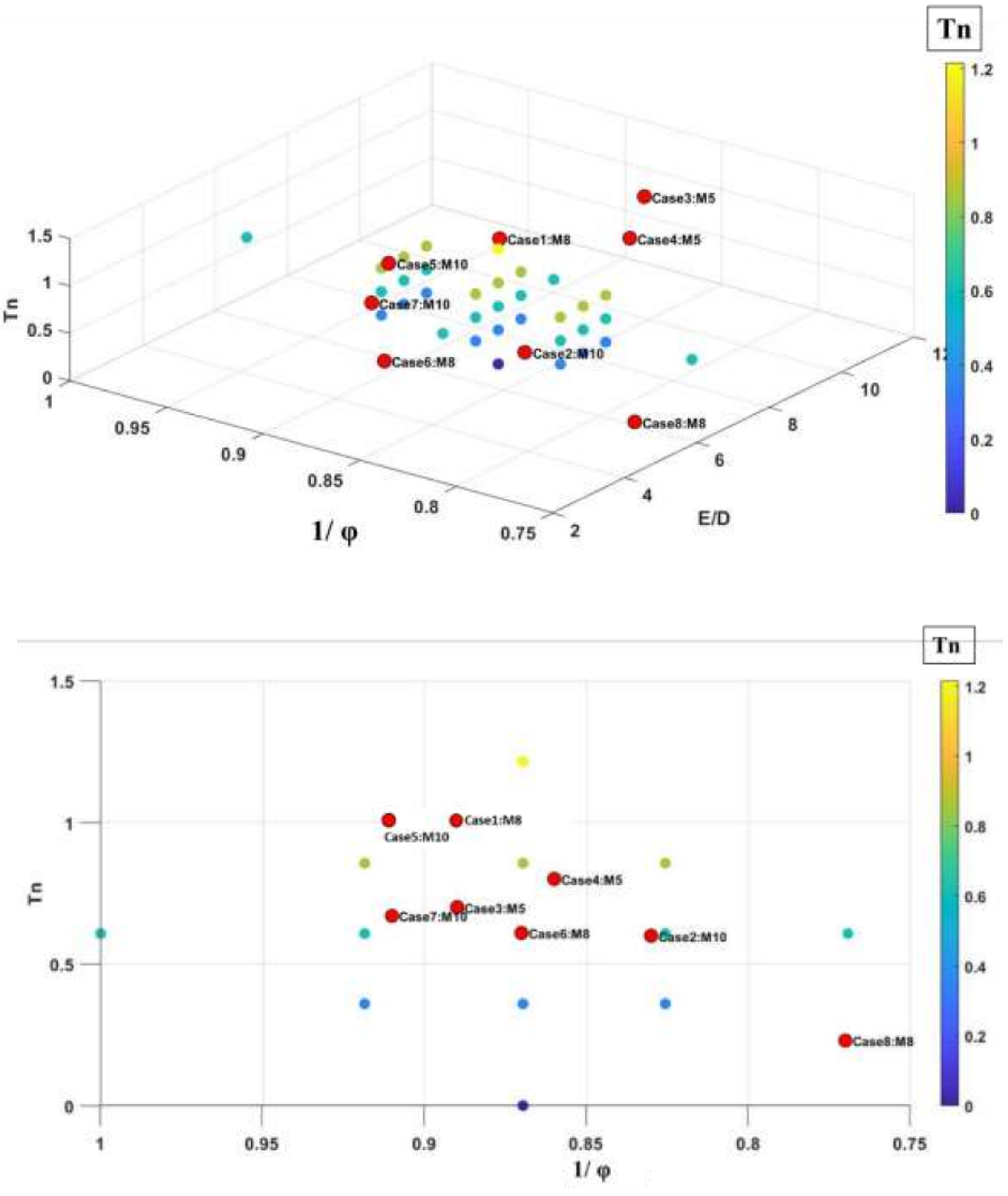

Fig. H2 3D scatter plot of W/D, E/D, and 1/ϕ with a 2D projection of 1/ϕ versus Tn, color-mapped by 1/ϕ, highlighting validation cases

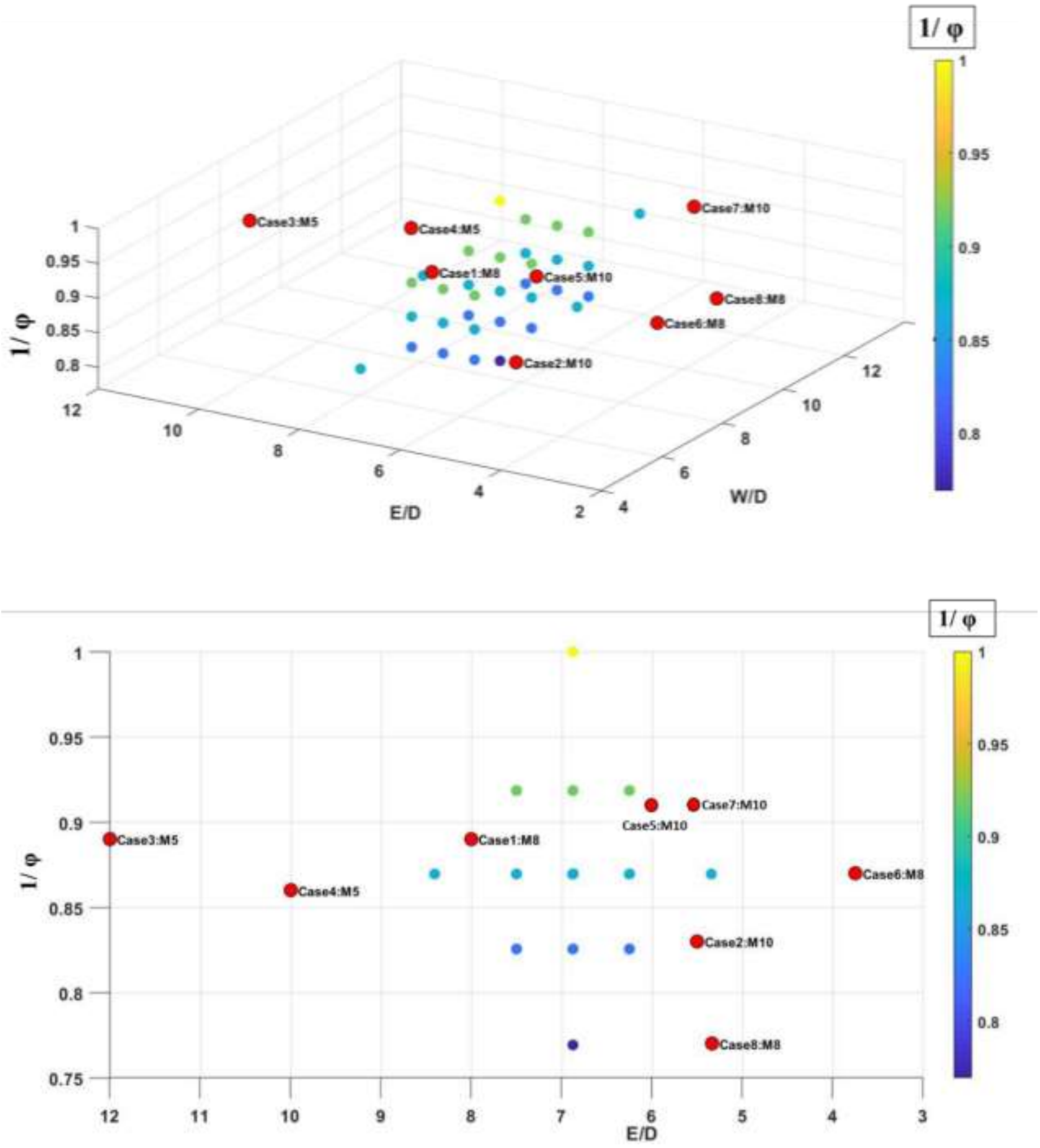

Fig. H3 3D scatter plot of E/D, 1/ϕ, and Tn with a 2D projection of E/D versus 1/ϕ, color-mapped by Tn, marking validation cases

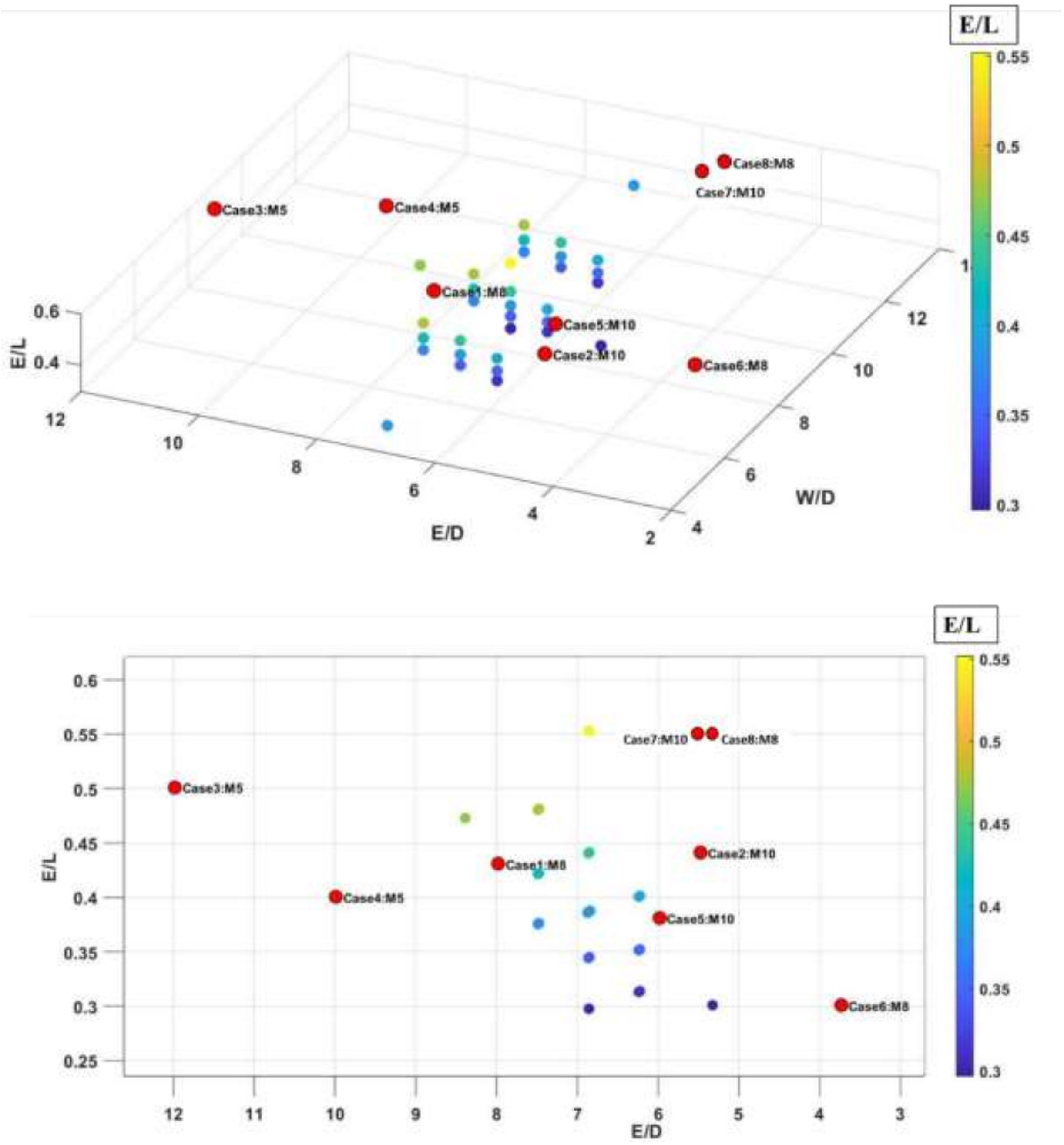

Fig. H4 3D scatter plot of W/D, E/D, and E/L with a 2D projection of E/D versus E/L, color-mapped by E/L, highlighting validation cases

Appendix I. Performance Metrics and Validation Results of ML Models

Table I1 RMSE and R^2 Values for Different Data Splits Based on Test Data

		LASSO Regression	Ridge Regression	Huber Regression	SVR
**70-30 Split**	RMSE	0.0360	0.0347	0.0353	0.0356
	R^2	0.9924	0.9929	0.9927	0.9925
**90-10 Split**	RMSE	0.0432	0.0381	0.0456	0.0443
	R^2	0.9853	0.9885	0.9836	0.9845

Table I2 Prediction Error Percentages for Models (70-30 Split)

	Percentage Error			
	**LASSO Regression**	**Ridge Regression**	**Huber Regression**	**SVR**
**Case 1**	0.25	0.55	0.41	2.57
**Case 2**	7.54	5.82	6.88	7.01
**Case 3**	**35.99**	**31.55**	7.49	**15.44**
**Case 4**	5.15	4.47	10.02	7.53
**Case 5**	7.68	7.23	7.74	7.35
**Case 6**	**16.14**	**15.69**	7.50	**10.73**
**Case 7**	6.62	3.77	7.27	7.77
**Case 8**	3.76	5.68	0.27	0.28
**Average Error**	*10.39*	*9.34*	*5.95*	*7.34*

Table I3 Prediction Error Percentages for Models (90-10 Split)

	Percentage Error			
	**LASSO Regression**	**Ridge Regression**	**Huber Regression**	**SVR**
**Case 1**	0.47	0.90	0.46	1.86
**Case 2**	9.46	5.76	7.09	7.49
**Case 3**	**32.66**	**19.80**	8.54	**11.53**
**Case 4**	9.40	4.48	8.18	5.65
**Case 5**	7.90	6.43	6.80	7.03
**Case 6**	**13.11**	**11.72**	8.01	8.42
**Case 7**	8.75	3.67	7.83	7.26
**Case 8**	2.19	7.06	0.80	1.88
**Average Error**	*10.49*	*7.48*	*5.96*	*6.39*

Appendix J. PySR Tuning Parameters

Table J1 Summary of PySR Parameters in Final Runs

**Number of islands**	31
**Population size per island**	1200
**Iterations per island**	1200
**Complexity**	23
**Unary operators**	Exponential and Sinusoidal
**Loss Function**	Log-Cosh Function

Appendix K. Performance Metrics, Prediction Errors, and Residual Analysis of PySR and Huber Models

Table K1 Comparison of RMSE and R² Values between Huber and PySR

	Huber Regression	PySR Expression
**RMSE**	0.0679	0.0630
**R^2**	0.9894	0.9908

Table K2 Prediction Error Percentages

	Percentage Error	
	**Huber Regression**	**PySR Expression**
**Case 1**	0.46	4.33
**Case 2**	7.09	5.84
**Case 3**	8.54	5.64
**Case 4**	8.18	6.18
**Case 5**	6.80	5.84
**Case 6**	8.01	4.04
**Case 7**	7.83	1.98
**Case 8**	0.80	3.46
**Average Error**	*5.96*	*4.66*

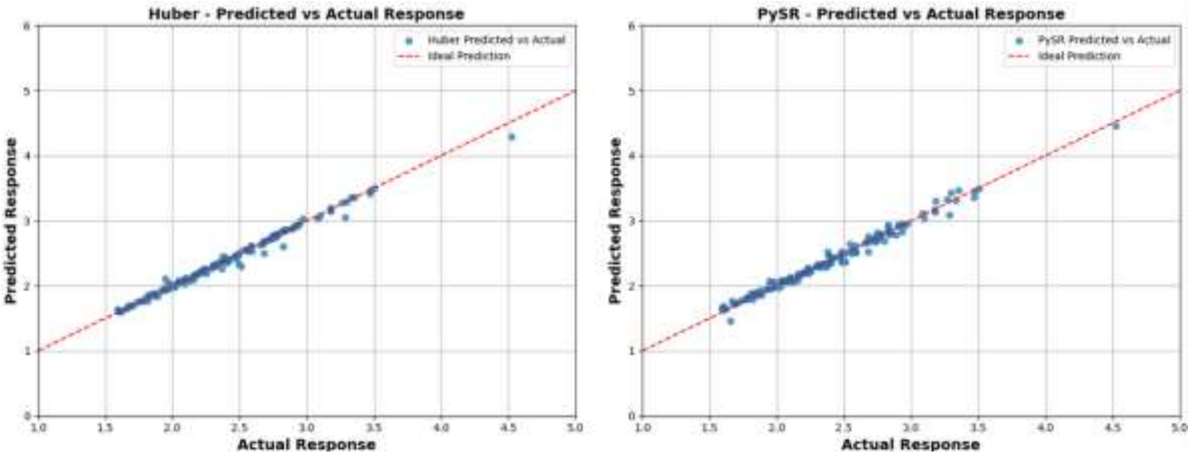

Fig. K1 Comparison of Predicted vs Actual Values Plots for Huber and PySR Models

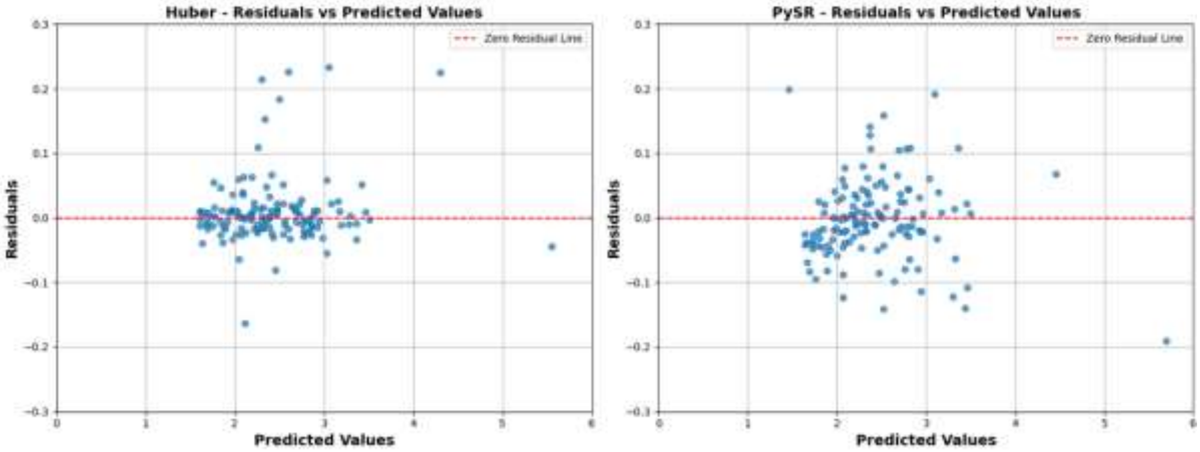

Fig. K2 Comparison of Predicted vs Residuals Plots for Huber and PySR Models

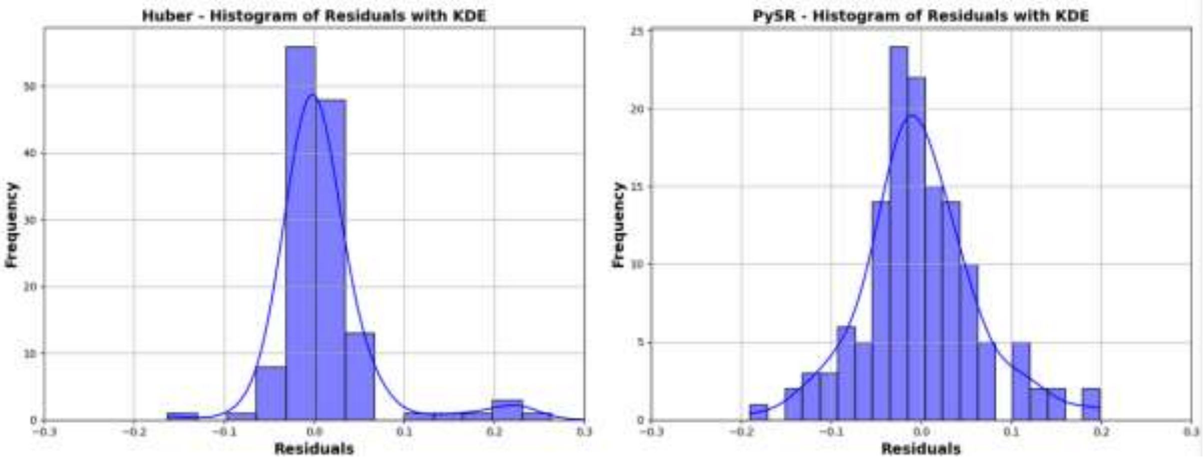

Fig. K3 Comparison of Residuals Distribution Plots for Huber and PySR Models

Appendix L. Validation Results for Multi-Bolt L-Joint Configurations

Table L1 Summary of Selected Cases and Relative Increase of Damage Initiation Load

	R/L	W/D	E/D	E/L	1/φ	Tn	Single Unit Damage Initiation Load (kN)	Repeated Unit Damage Initiation Load (kN)	Relative Increase
**Case 1**	0.12	8.375	8.375	0.46	**0.89**	**1.00**	2.54	2.64	1.04
**Case 2**	0.13	8.75	6.875	0.39	0.87	0.61	2.31	2.40	1.04
**Case 3**	0.17	9.00	**12.00**	0.50	0.89	0.70	2.51	2.51	1.00
**Case 4**	0.19	8.75	6.875	**0.55**	0.87	0.61	4.52	4.62	1.02
**Case 5**	**0.22**	8.75	6.875	0.39	0.87	0.61	2.38	2.50	1.05
**Case 6**	0.19	8.75	**3.75**	0.30	0.87	0.61	2.83	3.07	1.08
**Case 7**	0.10	8.75	6.875	**0.30**	0.87	0.61	1.60	1.63	1.02
**Case 8**	0.22	**13.34**	5.34	0.55	**0.77**	**0.23**	5.51	5.88	1.07
**Case 9**	0.13	**4.16**	6.875	0.39	0.87	0.61	1.49	1.57	1.05
**Case 10**	**0.05**	8.75	6.875	0.39	0.87	0.61	2.29	2.38	1.04

Table L2 Geometrical Representation for the Selected Cases

Case 1	Case 2	Case 3
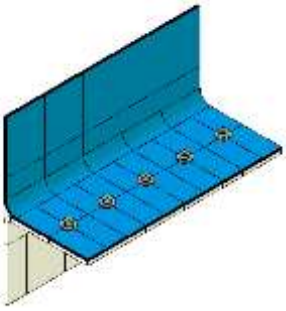	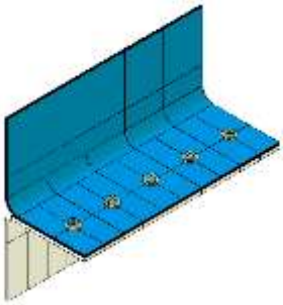	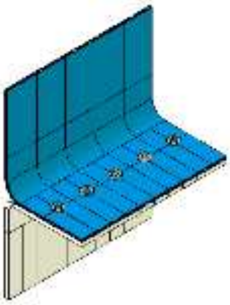
**Case 4**	**Case 5**	**Case 6**
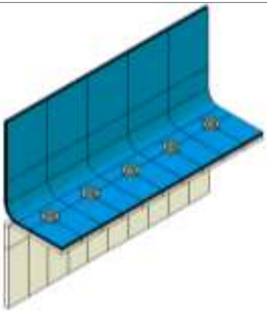	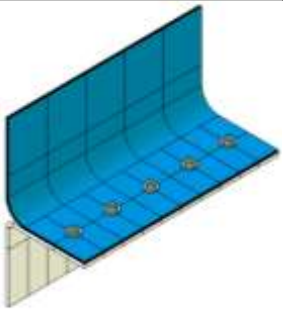	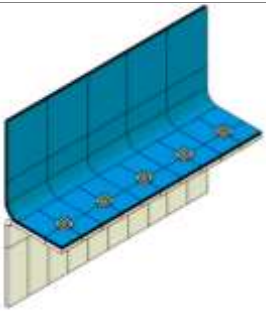
**Case 7**	**Case 8**	**Case 9**
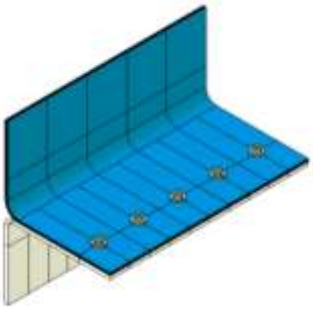	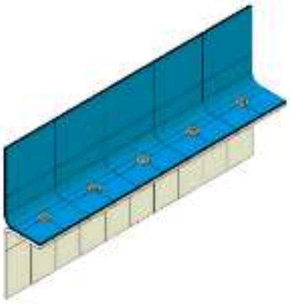	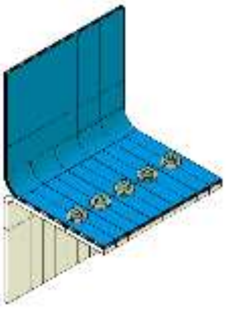
	**Case 10**	
	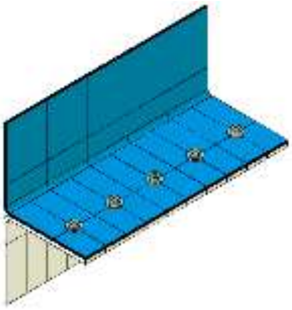	

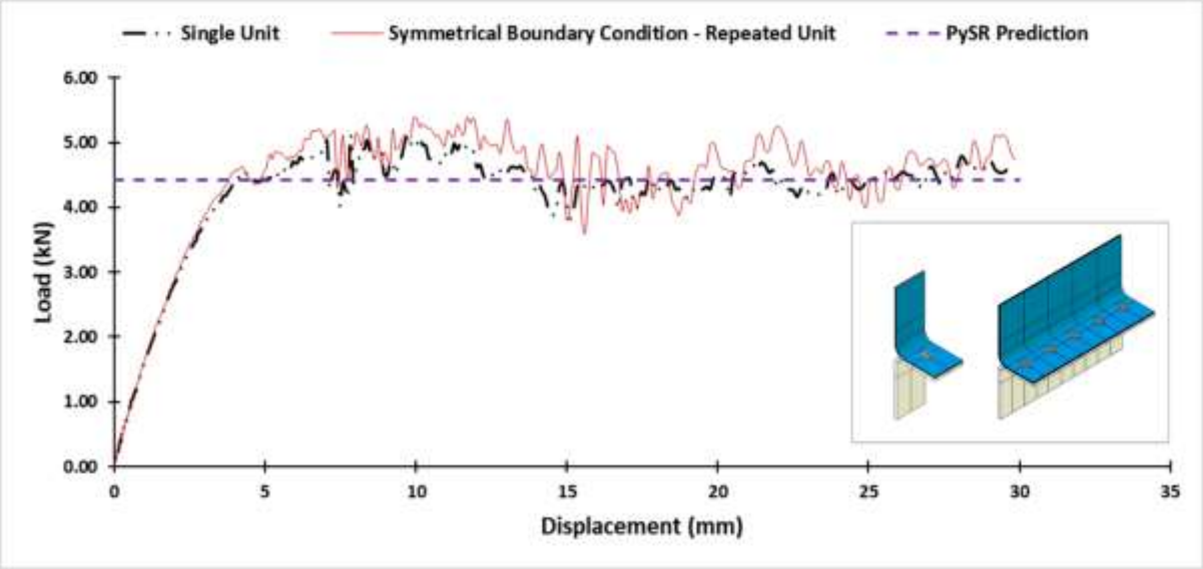

Fig. L1 Comparison of Load-Displacement Curves for Case 4 as a Single Unit and as a Repeated Unit, with PySR-Predicted Damage Initiation

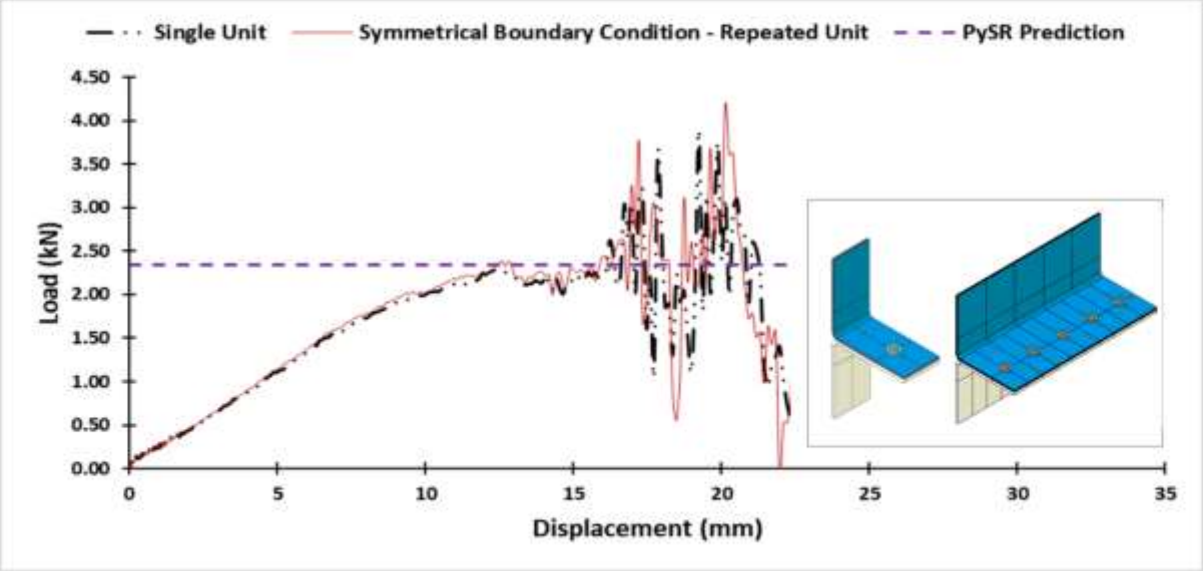

Fig. L2 Comparison of Load-Displacement Curves for Case 10 as a Single Unit and as a Repeated Unit, with PySR-Predicted Damage Initiation

Appendix M. Stacking Sequence Validation for PySR-Derived Equation

Table M1 Summary of Coefficients and Performance Metrics for Stacking Sequence [45/0/45/0]

Dataset Size	Coefficients		RMSE	R^2
**40 Simulations**	$a_1 = 0.5804$<br>$a_2 = -0.7690$	$c_1 = 3.9015$<br>$c_2 = 3.0746$<br>$c_3 = -1.5992$<br>$c_4 = -0.5653$	0.06855	0.9906

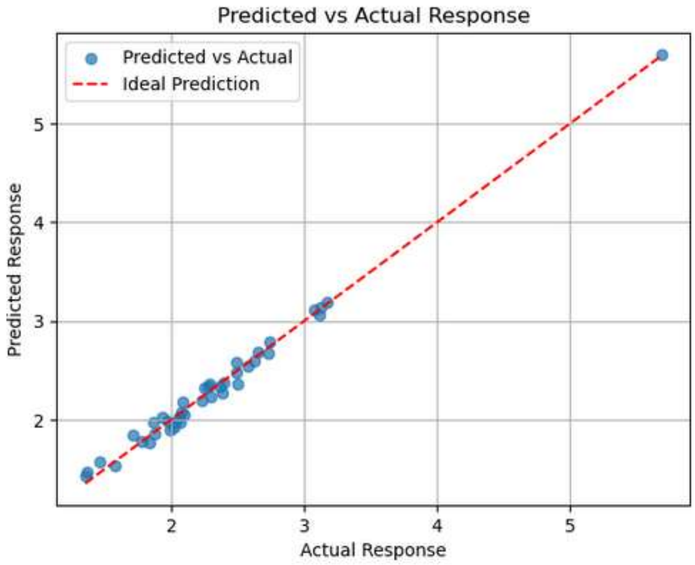

Fig. M1 Predicted vs Actual Values Plots for PySR Model Applied to the [45/0/45/0] Stacking Sequence

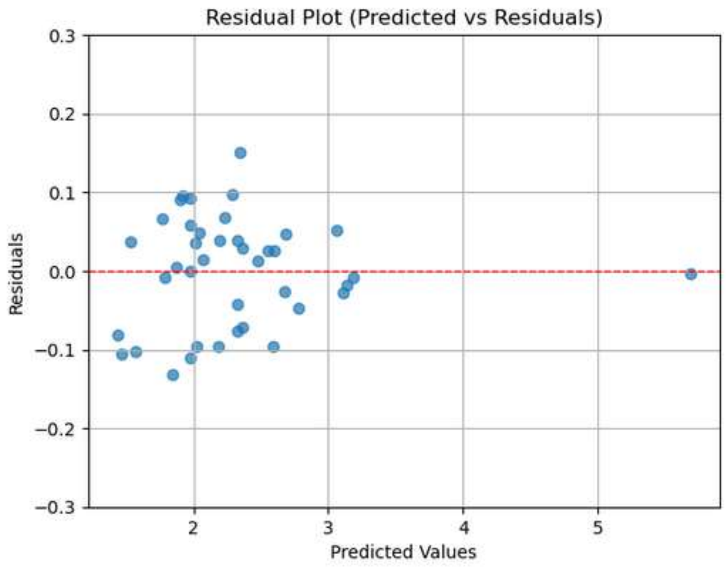

Fig. M2 Predicted vs Residuals Plot for PySR Model Applied to the [45/0/45/0] Stacking Sequence

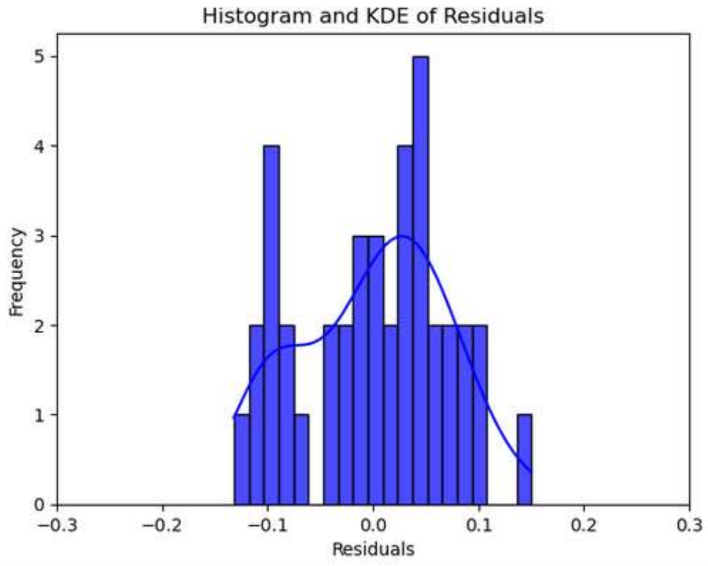

Fig M3 Residuals Distribution Plots for PySR Model Applied to the [45/0/45/0] Stacking Sequence
